# Supplementary material for: Computational and Experimental Evidence for Templated Macrocyclization: The Role of a Hydrogen Bond Network in the Quantitative Dimerization of 24-Atom Macrocycles
Source: Molecules. 2023 Jan 23;28(3):1144. doi: 10.3390/molecules28031144 (PMC9921993; doi:10.3390/molecules28031144)

SUPPORTING INFORMATION

Computational and Experimental Evidence for Templated Macrocyclization: The Role of a Hydrogen Bond Network in the Quantitative Dimerization to 24-Atom Macrocycles

Alexander J. Menke, Nicholas C. Henderson, Lola C. Kouretas, Anne N. Estenson, Benjamin G. Janesko* and Eric E. Simanek,*

[1]*Department of Chemistry & Biochemistry, Texas Christian University, Fort Worth TX 76129 USA*

AUTHOR CONTRIBUTIONS

AJM (Orchid ID# 0000-0001-9573-7045) – Led synthetic effort, prepared G-Acid, supervised undergraduate authors (ANE & LCK), coordinated data compilation, collected x-ray data, assembled SI and contributed significantly to the preparation of the manuscript.

NCH (Orchid ID# 0000-0002-5424-8349) – Contributed early efforts in computation.

LCK (Orchid ID# 0000-0003-0543-4118) – Synthesized $\mathbf{G}^{EA}$ and $\mathbf{V}^{EA}$.

ANE (Orchid ID# 0000-0002-2350-1903) – Synthesized $\mathbf{I}^{EA}$.

BGJ (Orchid ID# 0000-0002-2572-5273) – Designed, performed, and analyzed computation and supervised computation efforts.

EES (Orchid ID# 0000-0002-3195-4523) – Supervised project.

TABLE OF CONTENTS

I. **Chart S1.** Compounds described in the supporting material S3

II. General Experimental Details S4

III. Computational Figures S5

Figure S1. DFT-computed relative energies of protonated triazine models. S5
Figure S2. Coding the computational data onto the rotamer diagrams. S5
Figure S3. Relative energies of protonated triazine models. S6
Figure S4. Folding energy of the neutral (unprotonated) intermediate. S8
Figure S5. Folding energy of the monoprotonated (terminal) intermediate. S8
Figure S6. Folding energy of the monoprotonated (interior) intermediate. S9
Figure S7. Folding energy of the diprotonated intermediate. S9
Figure S8. Folding energy vs. number of protons. S10

IV. Experimental Details S11

V. Spectra S16
Figure S9. The 400 MHz ^{1}H NMR spectrum of **1** in DMSO-*d*$_6$. S17
Figure S10. The 100 MHz ^{13}C{^{1}H} NMR spectrum of **1** in DMSO-*d*$_6$. S18
Figure S11. The 400 MHz ^{1}H NMR spectrum of **2** in DMSO-*d*$_6$. S19
Figure S12. The 100 MHz ^{13}C{^{1}H} NMR spectrum of **2** in DMSO-*d*$_6$. S20
Figure S13. The 400 MHz ^{1}H NMR spectrum of **3** in DMSO-*d*$_6$. S21
Figure S14. The 100 MHz ^{13}C{^{1}H} NMR spectrum of **3** in DMSO-*d*$_6$. S22
Figure S15. The 400 MHz ^{1}H NMR spectrum of $\mathbf{G^{EA}}$ in DMSO-*d*$_6$. S23
Figure S16. The 100 MHz ^{13}C{^{1}H} NMR spectrum of $\mathbf{G^{EA}}$ in DMSO-*d*$_6$. S24
Figure S17. The 400 MHz COSY of $\mathbf{G^{EA}}$ in DMSO-*d*$_6$. S25
Figure S18. The 400 MHz ^{1}H NMR spectrum of $\mathbf{V^{EA}}$ in DMSO-*d*$_6$. S26
Figure S19. The 100 MHz ^{13}C{^{1}H} NMR spectrum of $\mathbf{V^{EA}}$ in DMSO-*d*$_6$. S27
Figure S20. The 400 MHz COSY of $\mathbf{V^{EA}}$ in DMSO-*d*$_6$. S28
Figure S21. The 400 MHz ^{1}H NMR spectrum of $\mathbf{I^{EA}}$ in DMSO-*d*$_6$. S29
Figure S22. The 100 MHz ^{13}C{^{1}H} NMR spectrum of $\mathbf{I^{EA}}$ in DMSO-*d*$_6$. S30
Figure S23. The 400 MHz COSY of $\mathbf{I^{EA}}$ in DMSO-*d*$_6$. S31
Figure S24. The 400 MHz ^{1}H NMR spectrum of **4** in DMSO-*d*$_6$. S32
Figure S25. The 100 MHz ^{13}C{^{1}H} NMR spectrum of **4** in DMSO-*d*$_6$. S33
Figure S26. The 400 MHz ^{1}H NMR of **G-G** in DMSO-*d*$_6$. S34
Figure S27. The 400 MHz ^{1}H NMR of **V-V** in DMSO-*d*$_6$. S35
Figure S28. The 400 MHz ^{1}H NMR of **I-I** in DMSO-*d*$_6$. S36

SECTION I

Chart S1. Compounds described in the supporting material

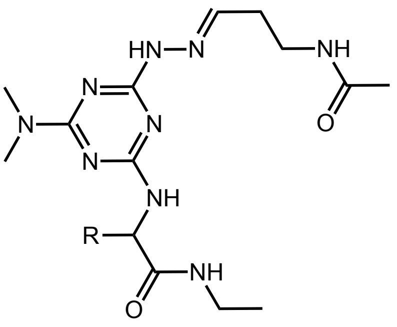

Name	R
$\mathbf{G^{EA}}$	H
$\mathbf{V^{EA}}$	$CH(CH_3)_2$
$\mathbf{I^{EA}}$	$CH(CH_3)(CH_2CH_3)$

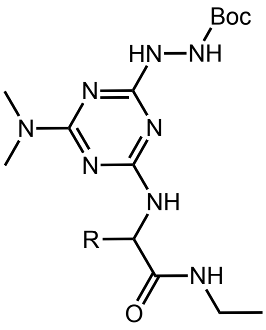

Name	R
**1**	H
**2**	$CH(CH_3)_2$
**3**	$CH(CH_3)(CH_2CH_3)$

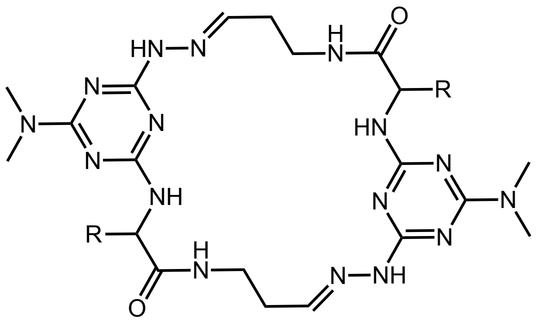

Name	R
**G-G**	H
**V-V**	$CH(CH_3)_2$
**I-I**	$CH(CH_3)(CH_2CH_3)$

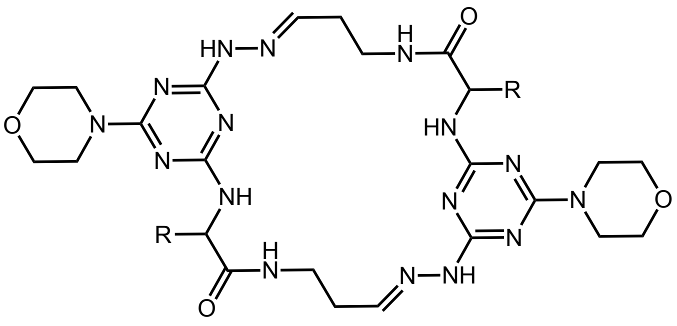

Name	R
**G'-G'**	H
**V'-V'**	$CH(CH_3)_2$
**I'-I'**	$CH(CH_3)(CH_2CH_3)$

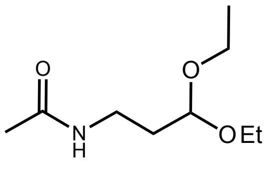

Acetal 4

SECTION II – General Experimental Details

NMR Spectroscopy. Room temperature ^{1}H NMR spectra were recorded on a 400 MHz Bruker Avance spectrometer. Chemical shifts for ^{1}H NMR spectra (in parts per million) referenced to a corresponding solvent resonance (e.g. DMSO-d_6, δ = 2.52 ppm). ^{13}C{^{1}H} NMR spectra were recorded on the same 400 MHz Bruker spectrometer referenced to corresponding solvent resonance. All 2D spectra were taken on the 400 MHz Bruker Avance relative to corresponding solvent resonances. Low temperature spectra were aquired on a 500 MHz Varian NMR spectrometer at the University of North Texas in Denton. Identification of NMR signals are as follows: s = singlet, d = doublet, t = triplet, dd = doublet of doublet, m = multiplet, etc. NMR solvents were deuterated and purchased as a bottle or ampule. Structural assignments were made with supported by two-dimensional NMR experiments (COSY, HSQC, and rOesy).

General Chemistry. Flash chromatography experiments were carried out on silica gel with a porosity of 60Å, particle size 50–63 m, surface area 500 – 600 m^2/g, a bulk density of 0.4 g/mL and a pH range of 6.5 –– 7.5. Dichloromethane/methanol was used as the eluent for chromatographic purification. Thin-layer chromatography experiments were carried out in sealed chambers and visualized with UV or submersion in ninhydrin (1.5g ninhydrin in 100mL of *n*-butanol and 3.0mL acetic acid) followed by heating. Excess solvents were removed via rotary evaporation on a Buchi Rotavapor RII with a Welch Self-Cleaning Dry Vacuum System. All workup and purification procedures were carried out with reagent-grade solvents under ambient atmosphere.

SECTION III – Computational Figures

Figure S1. DFT-computed relative energies of protonated triazine models. Calculations use M06-2X/6-311++G(2d,2p) geometry optimizations in SMD continuum water solvent. Each conformer is labeled with relative energy in kcal/mol.

Model Compounds to Assess Validity		
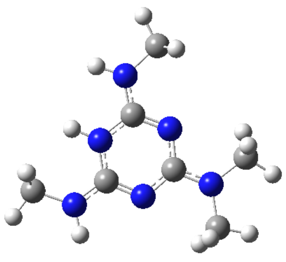 0.7 kcal/mol	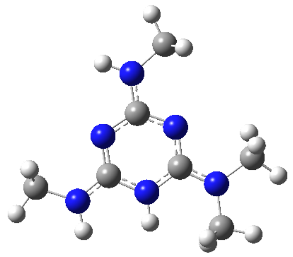 0.0 kcal/mol	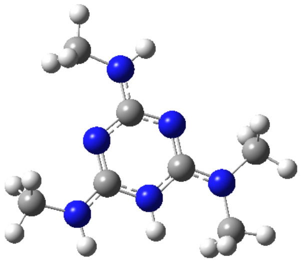 0.2 kcal/mol
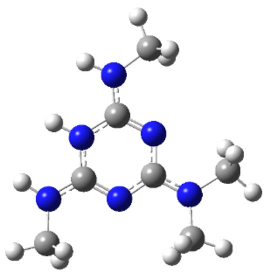 0.6 kcal/mol	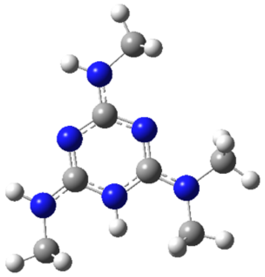 0.2 kcal/mol	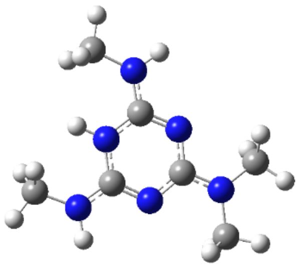 1.2 kcal/mol

Figure S2. Coding the computational data onto the rotamer diagrams. Dark blue is more energetically less costly than light blue. The data derives from Figure S1.

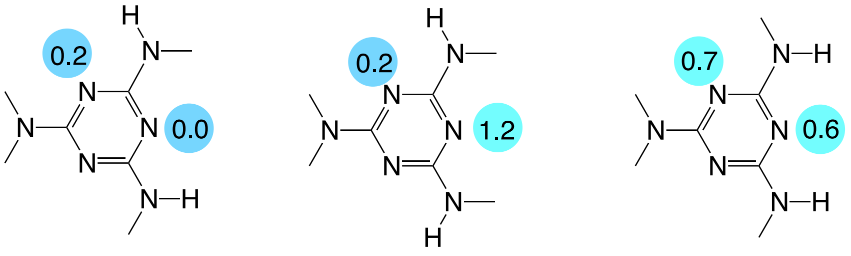

I or IV **II** **III**

Figure S3. Computed structures and protonation energies (Eq. 1) of protonated triazine models. Each conformer is labeled with the computed reaction energy of Eq. 1 in kcal/mol.

Hydrazones of Interest		
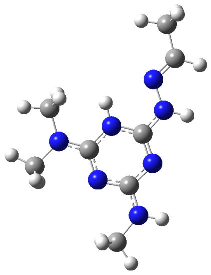<br>0.0 kcal/mol (IIc)	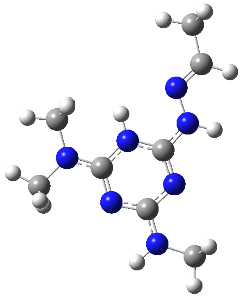<br>0.2 kcal/mol (IIIc)	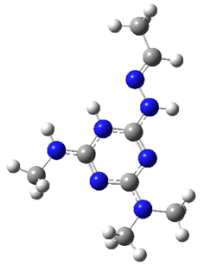<br>0.9 kcal/mol (Ia)
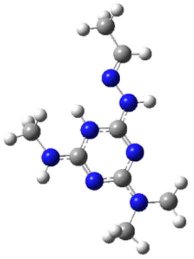<br>0.9 kcal/mol (IVa)	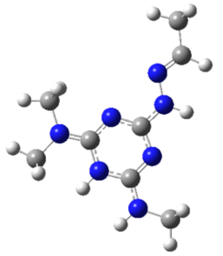<br>1.5 kcal/mol (IIIb)	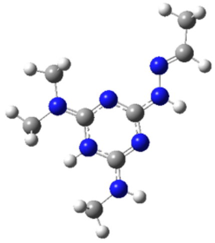<br>1.6 kcal/mol (IIb)
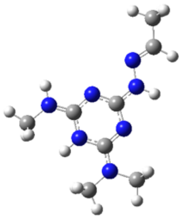<br>1.8 kcal/mol (Ib)	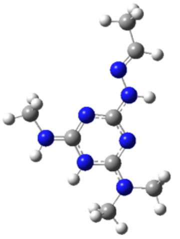<br>1.6 kcal/mol (IVb)	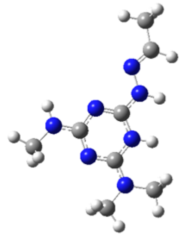<br>3.0 kcal/mol (Ic)
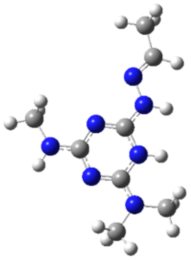<br>3.2 kcal/mol (IVc)	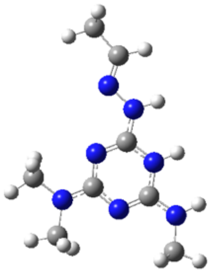<br>3.4 kcal/mol (IIa)	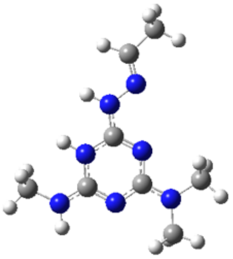<br>3.6 kcal/mol (IIIa)

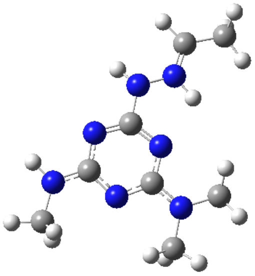 9.5 kcal/mol (IId)	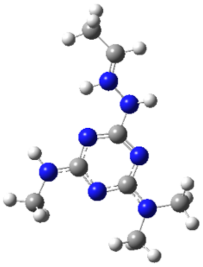 9.8 kcal/mol (Id)	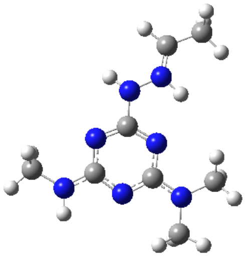 9.7 kcal/mol (IIId)
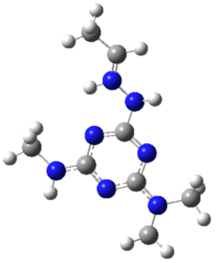 9.9 kcal/mol (IVd)		

Figure S4. Folding energy of the neutral (unprotonated) intermediate. The DFT-computed relative energies in Figures S4-S8 were calculated using M06-2X/6-31+G(d) with the SMD continuum model for water solvent. Several optimizations converged to partially folded states. For reference, only the "most folded" and the "completely unfolded" conformations are shown. Hydrogen bonds are drawn as a guide to the eye. In all cases, the completely unfolded dimer is higher in energy ($\Delta U>0$). The value of ΔS is not computed. For the neutral (unprotonated) intermediate, the values for the folded and unfolded conformations are 0 kcal/mol and +6.4 kcal/mol, respectively.

**Folded**	**Unfolded**
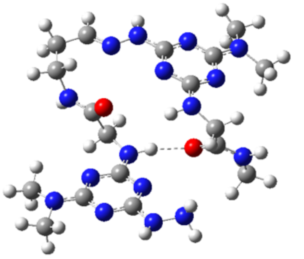	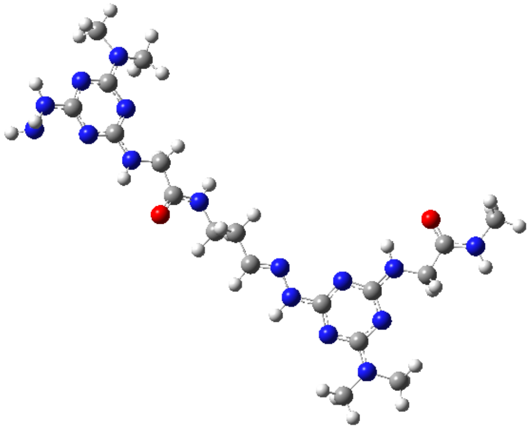

Figure S5. Folding energy of the monoprotonated (terminal) intermediate. The values for the folded and unfolded conformations are 0 kcal/mol and +10.1 kcal/mol, respectively.

**Folded**	**Unfolded**
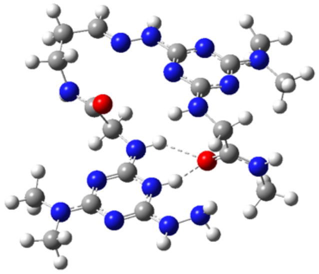	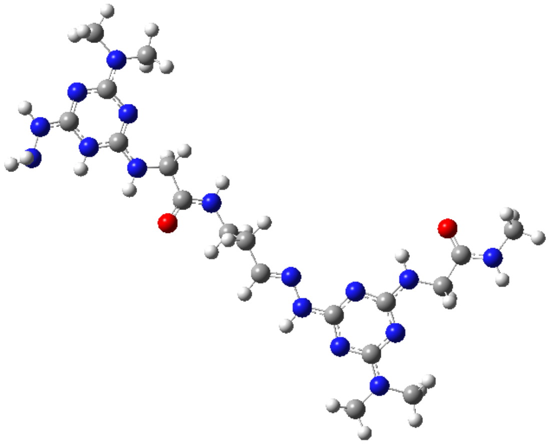

Figure S6. Folding energy of the monoprotonated (interior) intermediate. The values for the folded and unfolded conformations are 0 kcal/mol and +12.4 kcal/mol, respectively.

Folded	Unfolded
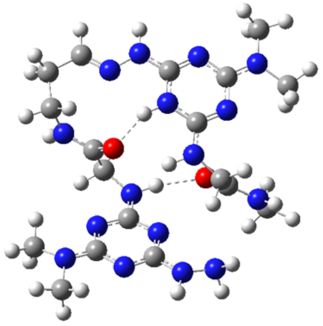	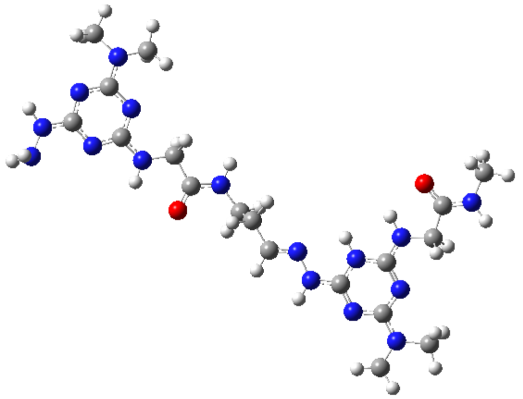

Figure S7. Folding energy of the diprotonated intermediate. The values for the folded and unfolded conformations are 0 kcal/mol and +14.2 kcal/mol, respectively.

Folded	Unfolded
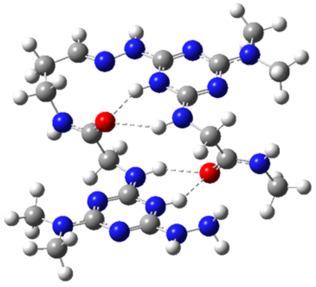	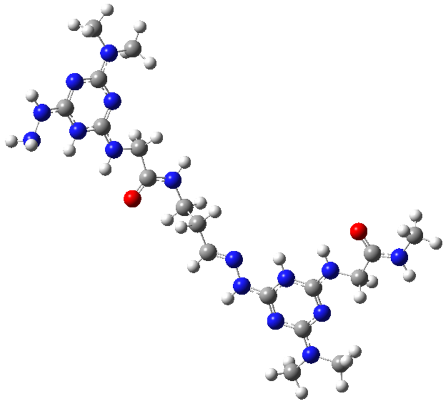

Figure S8. Folding energy vs. number of protons. The plot is reasonably linear with a slope of 3.9 kcal/mol folding energy per added proton. "Templating" is predicted to be increasingly important at low pH. Presumably, the selectivity to dimer cyclization is also improved at low pH.

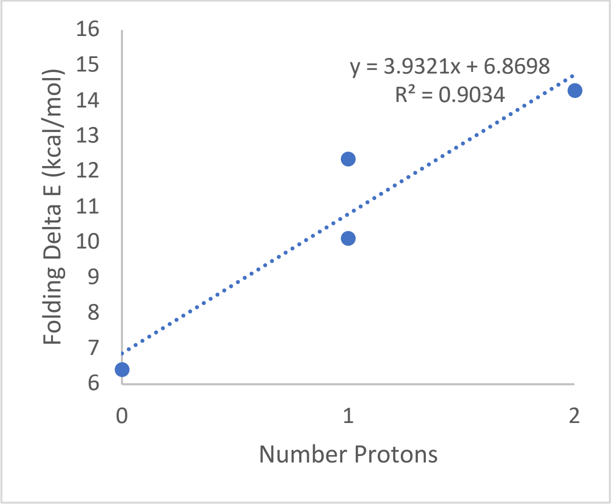

SECTION IV – Experimental Details

Synthesis of R-Acid

Acid intermediates were prepared via a previously published route. The method for **G-Acid** is shown below as an example.

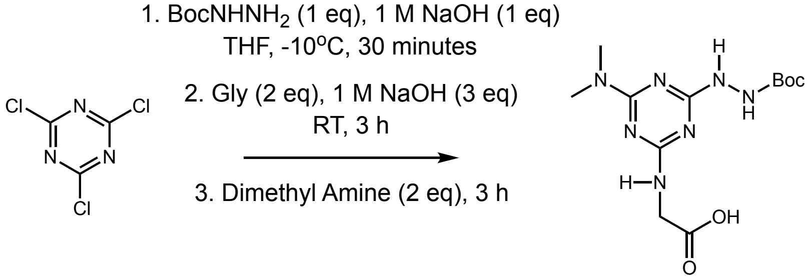

Cyanuric chloride (1.0 g, 5.4 mmol) was added rapidly as a solid to a stirring flask containing 15 mL of THF that was previously cooled to –10 °C using a dry ice and acetone bath. The temperature was maintained at –10 °C for the duration of the reaction. Upon dissolution (which was immediate), a 3 mL solution of BOC-hydrazine (0.71 g, 5.4 mmol) in THF (1.8 M) was added dropwise over 5 minutes. Over the course of the addition, the solution turned a very pale yellow. After the addition was complete 5.4 mL of 1 M NaOH (5.4 mmol) was added over 1 minute via pipette. After one hour, thin layer chromatography (10% Methanol in Dichloromethane) showed the evolution of a single UV active spot with a retention factor of 0.7.

A solution of glycine (0.81 g, 10.8 mmol) in 5 mL H_2O and 16.2 mL of 1 M NaOH was added dropwise over 2 min while at room temperature. The solution started a pale yellow and turned bronze in color. After 4.25 h, thin layer chromatography (10% MeOH in DCM) showed the starting material (R_f = 0.7) disappeared and a new spot at R_f = 0.0 appeared using both short wave UV irradiation or ninhydrin (yellow spot).

A 40% aqueous solution of dimethylamine (1.83 g, 16.2 mmol) was added dropwise over three minutes. Immediately following addition, the solution was measured to be pH 9. The reaction was allowed to stir for 3 h before removing excess solvent via air stream as the product is sufficiently hydrophilic. The resulting white residue was washed 5 times with MeOH (~150 mL) and filtered before drying over $MgSO_4$. Crude product was taken forward.

Characterization data can be found in:

Capelli, R.; Menke, A. J.; Pan, H.; Janesko, B. G.; Simanek, E. E.; Pavan, G. M. Well-Tempered Metadynamics Simulations Predict the Structrual and Dynamic Properties of a Chiral 24-atom Macrocycle in Solution. *ACS Omega*, **2022**, *7*, 34, 30291-30296.

Synthesis of Ethyl Amide Intermediates

Synthesis of the amide intermediates use the general procedure seen here. Further details for each species can be seen below. One equivalent of the respective acid intermediate was dissolved in either DCM or DMF (0.15 M) at room temperature. Each reagent was added to the reaction neat as either a solid or liquid. In the case of a liquid the reagent was dropped in by pipette over 1 minute. The order of addition follows as DIPEA first, followed by the respective coupling agent, and a 66% solution of ethyl amine. The reaction was allowed to react for a minimum of 4 hours as determined by TLC analysis. Once completed, the reaction mixture is diluted with water and washed repeatedly with water three times. After extraction, column chromatography was utilized to purify the crude reaction product.

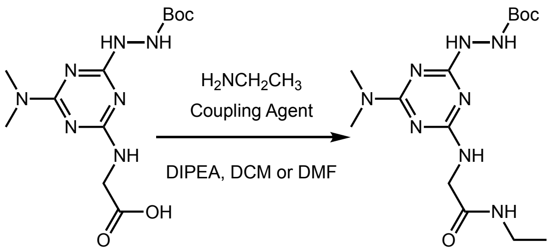

Synthesis of 1. Crude **G-Acid** (0.5 g, 1.5 mmol) was dissolved in 10 mL of DMF (0.1 M) and DIPEA (3.75 mmol) was added dropwise. Following this addition, EDC.HCl (1.8 mmol) was added as a solid to the reaction mixture, followed by a 66% solution of ethylamine (1.8 mmol) dropwise. The reaction was allowed to stir overnight before drying down via an airstream. Column chromatography in 5% MeOH in DCM was used to purify the crude reaction to yield 0.077 g (15%) as a white solid.

^{1}H NMR (DMSO-D$_6$, 400 MHz): δ 8.48 – 8.05 (m, 2H), 7.72 (brd s, 1H), 6.74 (m, 1H), 3.85 – 3.74 (m, 2H), (3.08 (m, J = 6.3 Hz, 2H), 3.01 (brd s, 6H), 1.40 – 1.24 (m, 9H), 1.00 (t, J = 6.3 Hz, 3H)
^{13}C{^{1}H} NMR (DMSO-D$_6$, 100 MHz): δ 169.9, 167.9, 166.4, 165.8, 156.5, 79.0, 44.4 – 44.0, 35.8, 31.2, 28.6, 15.3.

Synthesis of 2. V-Acid (1.4 mmol) was dissolved in DCM (0.2 M) and DIPEA (3.5 mmol) was added neat, dropwise over one minute. Following this, HOBT (1.7 mmol), HBTU (1.7 mmol), and a 66% solution of ethylamine (1.4 mmol) were added to the reaction. The reaction was allowed to react overnight before performing column chromatography on the crude product without extraction. The column was run with 5% MeOH in DCM allowing 0.127 g of pure product (23%) as a colorless oil.

^{1}H NMR (DMSO-D$_6$, 400 MHz): δ 8.50 (s, 1H), 8.31 – 8.20 (m, 1H), 7.81 – 7.55 (m, 1H), 6.33 – 6.23 (m, 1H), 4.58 – 4.21 (m, 1H), 3.08 (q, J = 8 Hz, 2H), 3.02 (s, 6H), 1.80 (broad s, 1H), 1.41 (s, 9 H), 1.30 (broad s, 2H), 1.00 (t, J = 8 Hz, 3H), 0.83 (m, 6H).
^{13}C{^{1}H} NMR (DMSO-D$_6$, 100 MHz): δ 171.5, 167.3, 165.3, 156.0, 78.5, 59.7, 35.4, 33.3, 30.4, 28.2, 19.4, 14.7.

Synthesis of 3. I-Acid (0.8 mmol) was dissolved in DCM (0.1 M) and DIPEA (0.8 mmol) was added neat, dropwise over one minute. Following this, HOBT (0.8 mmol), HBTU (0.8 mmol), and a 66% solution of ethylamine (0.8 mmol) were added to the reaction. The reaction was allowed to react for 36 hours before performing column chromatography on the crude product without extraction. The column was ran with 5% MeOH in DCM allowing 0.160 g of pure product (49%) as a colorless oil.

^{1}H NMR (DMSO-D_6, 400 MHz): δ 8.50 (s, 1H), 8.31 – 8.20 (m, 1H), 7.81 – 7.55 (m, 1H), 6.33 – 6.23 (m, 1H), 4.58 – 4.21 (m, 1H), 3.08 (q, J = 8 Hz, 2H), 3.02 (s, 6H), 1.80 (broad s, 1H), 1.41 (s, 9 H), 1.30 (broad s, 2H), 1.00 (t, J = 8 Hz, 3H), 0.83 (m, 6H).
^{13}C{^{1}H} NMR (DMSO-D_6, 100 MHz): δ 172.3, 167.9, 165.8, 156.5, 79.0, 58.9 – 57.4, 37.3, 35.8, 33.9, 28.6, 26.3, 21.5, 16.0, 15.2, 12.2, 12.1, 11.5.

Synthesis of 4

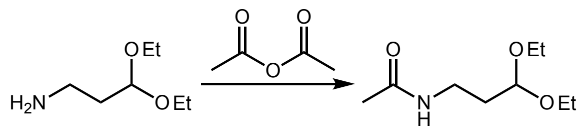

Acetic anhydride (0.257 g, 2.52 mmol) was added to a round bottom flask and cooled to -10 °C. To the flask, (3,3-diethyoxypropyl) amine (0.370 g, 2.52 mmol) was added over 15 minutes. The reaction was stirred for 1 hour before adding 10 mL of toluene and evaporating to dryness to yield 0.465 g (97%) pure product as a brown oil. NMR shows a 7:3 mixture of acetal and aldehyde which are recovered in 100% yield. The aldehyde is indicated by resonances occurring at 9.64 and 4.44. The following chemical shifts relate to the major product.

^{1}H NMR (DMSO-D$_6$, 400 MHz): δ 7.78 (s, 1H), 4.49 (t, J = 11.3, 1H), 3.56 (m, 2H), 3.42 (m, 2H), 3.02 (m, 2H), 1.78 (s, 3H), 1.64 (m, 2H), 1.11 (t, J = 6.6, 6H).
^{13}C{^{1}H} NMR (DMSO-D$_6$, 100 MHz): δ 202.8, 100.9, 61.1, 43.8, 33.3, 22.9, 15.8.

Synthesis of Hydrazone Models

G^{EA}, **V^{EA},** and **I^{EA}** were all synthesized via similar synthetic procedures. For **G^{EA}**, **V^{EA},** and **I^{EA}**, molar equivalents of **4** and the respective ethyl amide intermediate are added to a 3 mL vial with 0.5 mL of dichloromethane equipped with a mini-stir bar. Once both materials were added to the vial (totaling roughly 1 mL DCM), 1 mL of trifluoroacetic acid was added dropwise with stirring. Once TFA was added, the reaction was allowed to slowly evaporate for 1-3 days before taking NMRs. The final product was a brown oil in all cases.

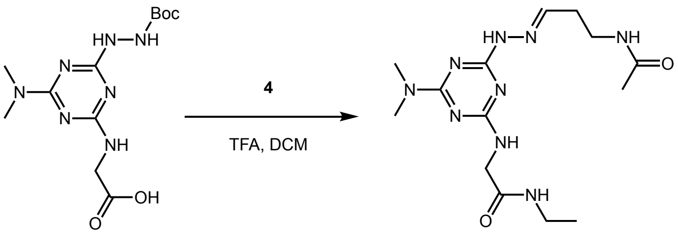

G^{EA}

^{1}**H NMR** (DMSO-D$_6$, 400 MHz): δ 12.35 (s, 1H), 11.65 (broad s, 1H), 8.16 (q, J = 4.4 Hz, 1H), 8.03 (t, J = 6.4 Hz, 1H), 7.56 (t, J = 5.2 Hz, 1H), 4.01 (d, J = 4.4 Hz, 2H), 3.30 (q, J = 6.4 Hz, 2H), 3.17 (s, 3H), 3.13 (s, 3H), 3. 12 – 3.10 (m, J = 7.3 Hz, 2H), 2.45 (q, J = 5.2 Hz, 2H), 1.81 (s, 3H), 1.04 (t, J = 7.3 Hz, 3H). **^{13}C{^{1}H} NMR** (DMSO-D$_6$, 100 MHz): δ 169.9, 167.4, 154.4, 154.9, 151.6, 43.7, 36.9, 36.1, 34.0, 33.2, 23.1, 15.1.

V^{EA}

^{1}**H NMR** (DMSO-D$_6$, 400 MHz): δ 12.37 (s, 1H), 11.42 (broad s, 1H), 8.29 (t, J = 5.6 Hz, 1H), 8.20 (d, J = 8.0 Hz, 1H), 8.05 (t, J = 5.0 Hz, 1H), 7.56 (t, J = 5.3 Hz, 1H), 4.42 (dd, J = 8.0, 5.7 Hz, 1H), 3.30 (q, J = 5.6 Hz, 2H), 3.17 – 3.06 (m, 8H), 2.45 (q, J = 5.0 Hz, 2H), 2.07 (m, 1H), 1.8 (s, 3H), 1.04 (t, J = 7.2 Hz, 3H), 0.91 (dd, J = 11.5, 7.0 Hz, 6H). **^{13}C{^{1}H} NMR** (DMSO-D$_6$, 100 MHz): δ 169.6, 169.3, 161.8, 153.9, 153.4, 151.2, 58.7, 38.3, 36.6, 35.7, 33.5, 32.9, 31.3, 26.6, 19.2, 14.7.

I^{EA}

^{1}**H NMR** (DMSO-D$_6$, 400 MHz): δ 12.38 (s, 1H), 11.42 (broad s, 1H), 8.27 (q, J = 8 Hz, 1H), 8.21 (dd, J = 24 Hz, 12 Hz, 1H), 8.05 (m, J = 4 Hz, 1H), 7.55 (td, J = 8 Hz, 4 Hz, 1H), 4.55 (dd, J = 8 Hz, 4 Hz, 1H), 4.41 (dd, J = 8 Hz, 4 Hz, 1H), 3.30 (q, J = 8 Hz, 2H), 3.18 (s, H), 3.17 (s, H), 3.13 (s, 3H), 3.11 – 3.05 (m, 2H), 2.46 (qd, J = 8 Hz, 4 Hz, 2H), 1.91 – 1.83 (m, 2H), 1.81 (s, 3H), 1.55 – 1.43 (m, 1H), 1.42 – 1.34 (m, 1H), 1.23 – 1.08 (m, 2H), 1.03 (td, J = 8 Hz, 0.8 Hz, 3H), 0.92 – 0.85 (m, 6H). **^{13}C{^{1}H} NMR** (DMSO-D$_6$, 100 MHz): δ 170.0, 169.9, 162.3, 154.4, 154.1, 154.8, 151.6, 151.5, 58.4, 57.3, 43.7, 38.1, 36.9, 33.9, 33.3, 33.0, 26.3, 24.7, 23.0, 15.0, 14.7, 12.1, 11.7.

SECTION V – Spectra

Figure S9. The 400 MHz ^{1}H NMR spectrum of **1** in DMSO-d_6.

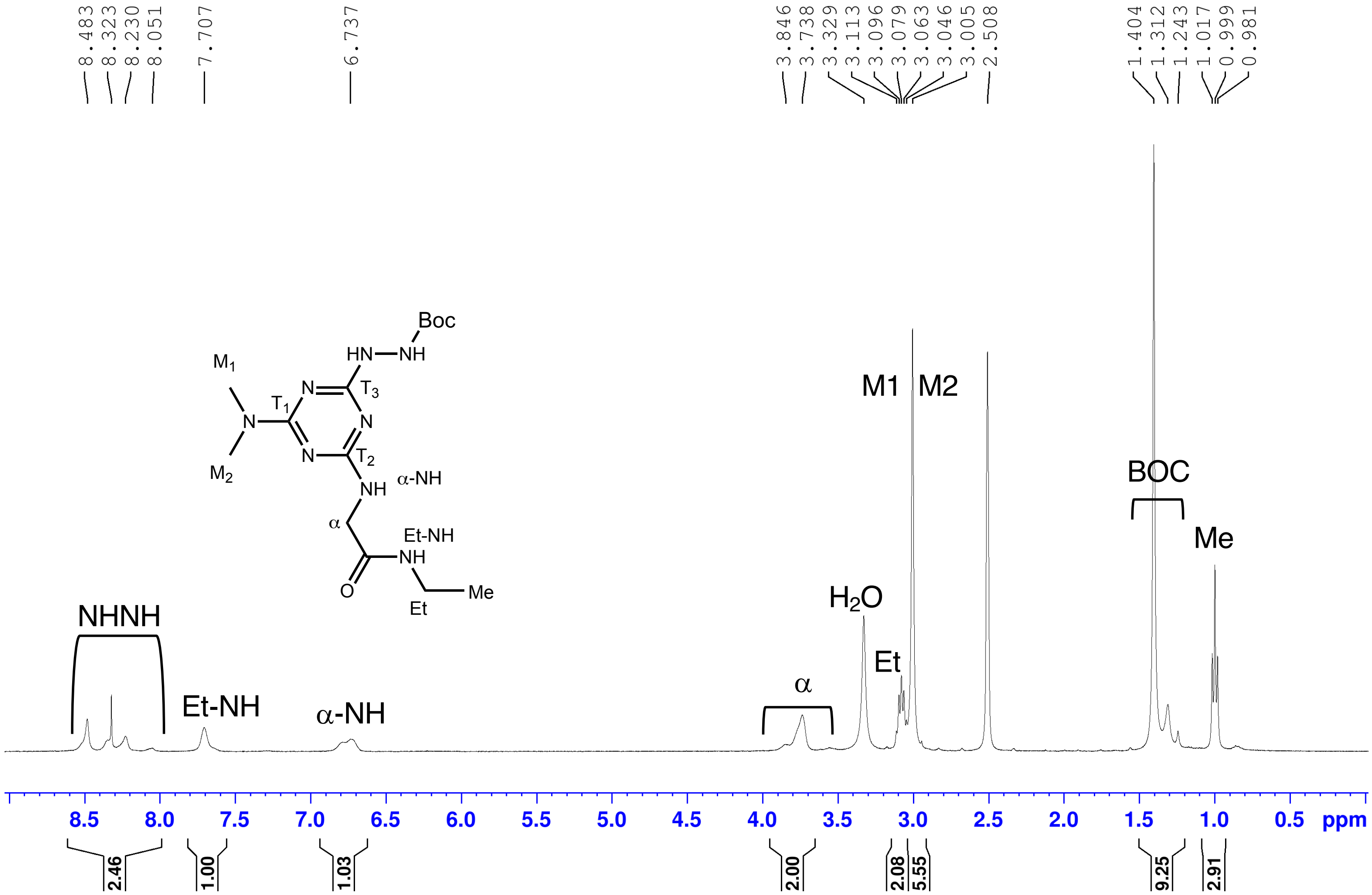

Figure S10. The 100 MHz $^{13}C\{^1H\}$ NMR spectrum of **1** in DMSO-d_6.

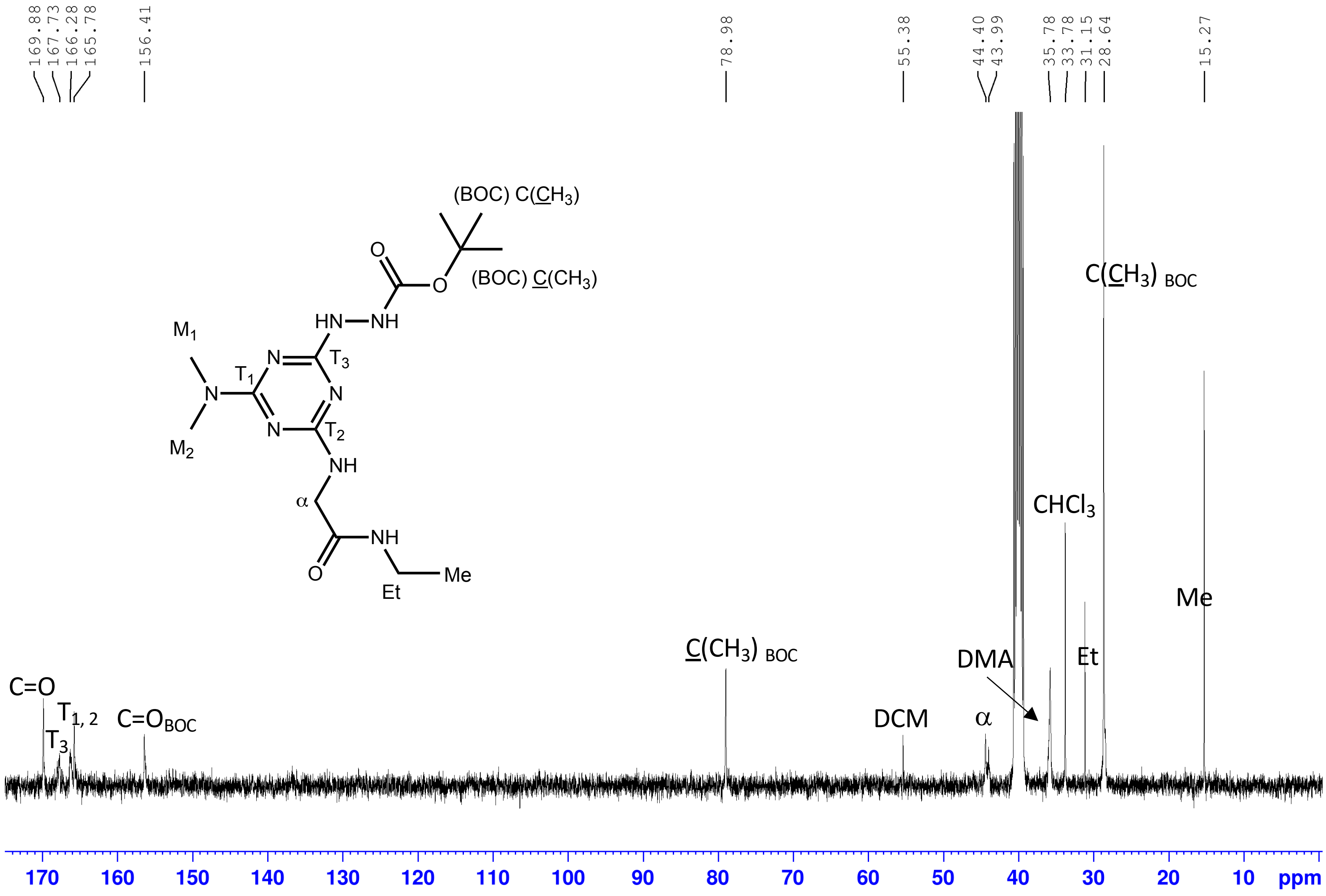

Figure S11. The 400 MHz ^{1}H NMR spectrum of **2** in DMSO-d_6. Spectra for precursors appears in a separate manuscript currently under review.

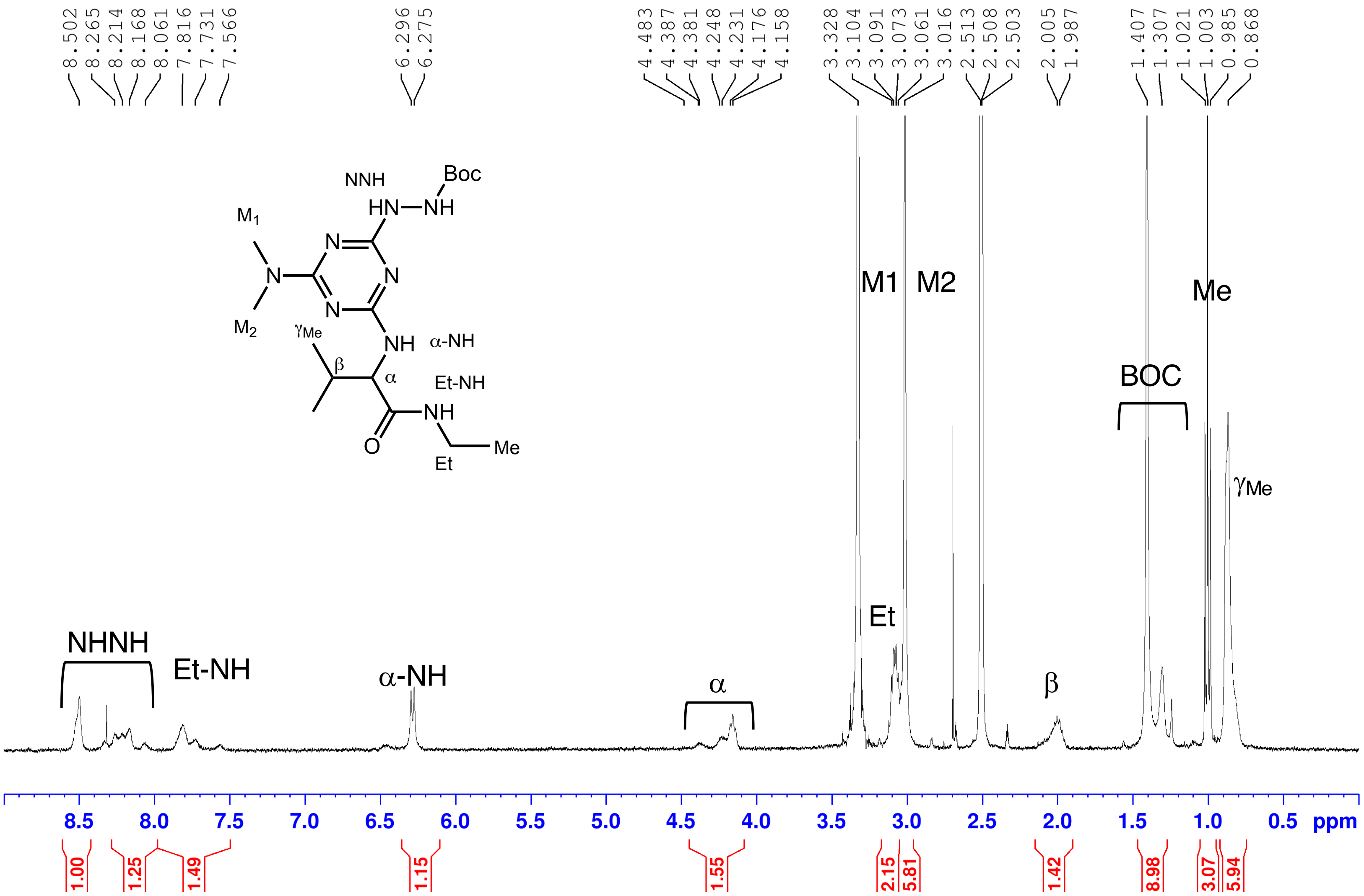

Figure S12. The 100 MHz $^{13}C\{^1H\}$ NMR spectrum of **2** in DMSO-d_6.

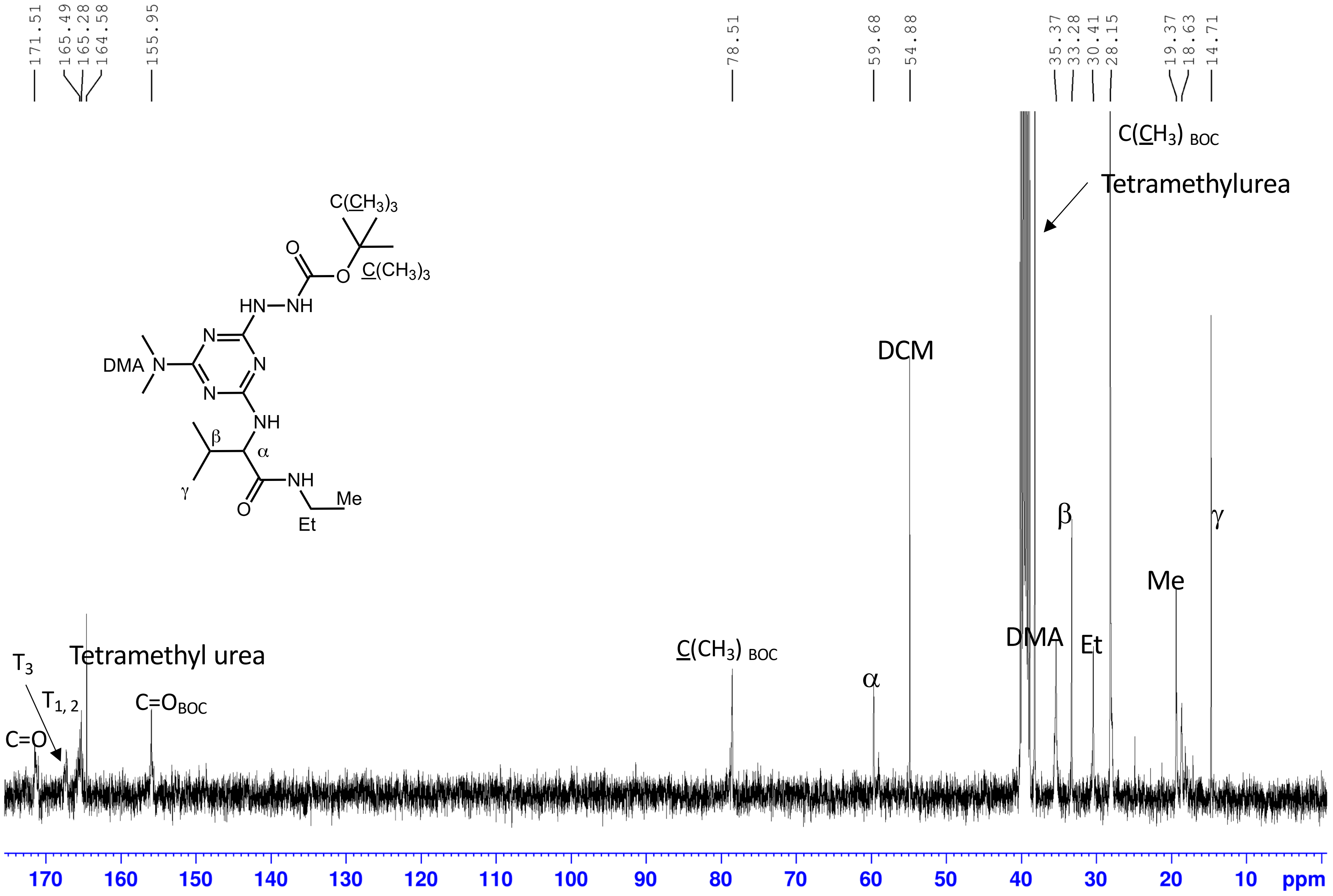

Figure S13. The 400 MHz ^{1}H NMR spectrum of **3** in DMSO-d_6.

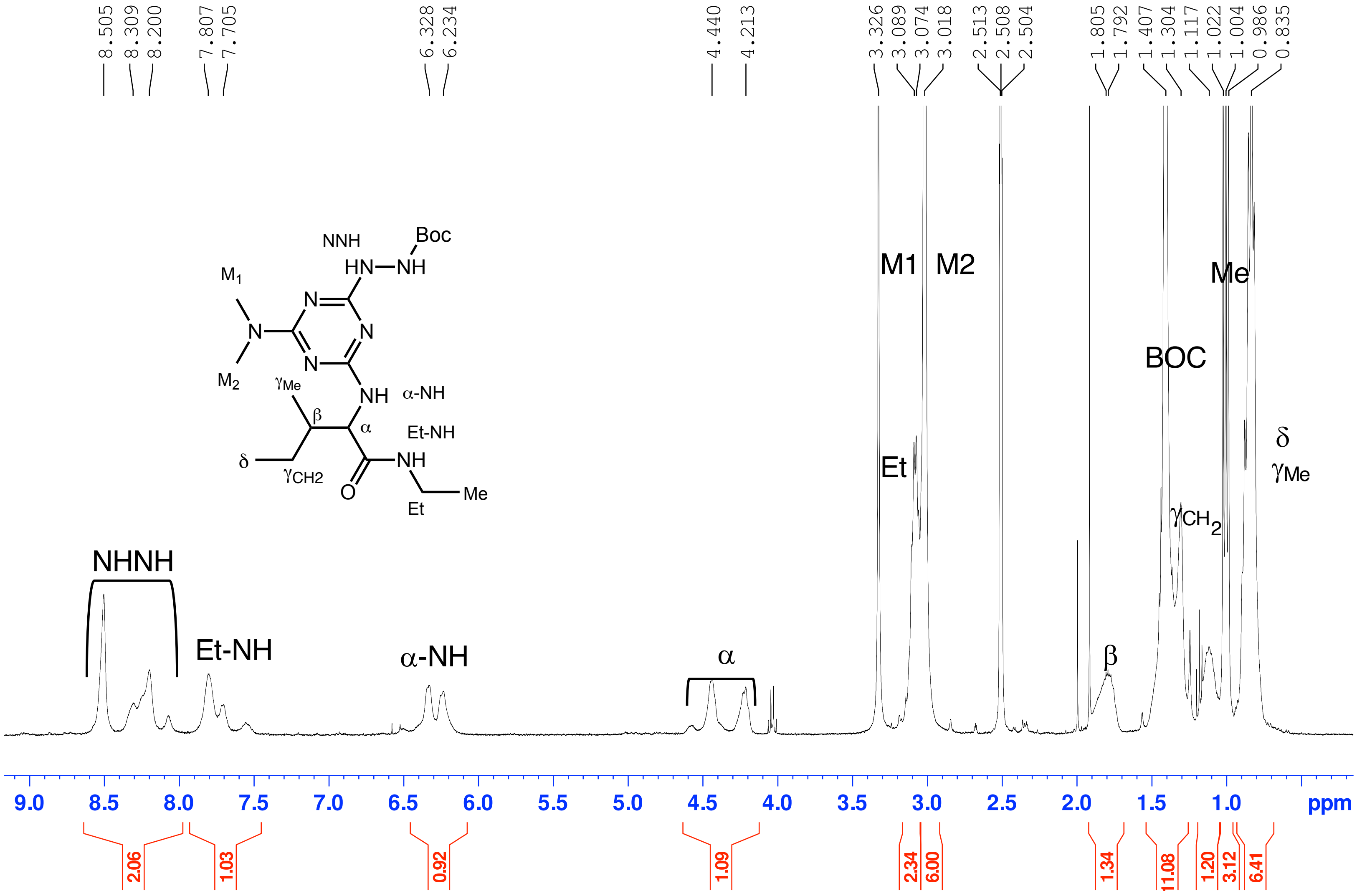

Figure S14. The 100 MHz $^{13}C\{^1H\}$ NMR spectrum of **3** in DMSO-d_6. Spectra for precursors appears in a separate manuscript currently under review.

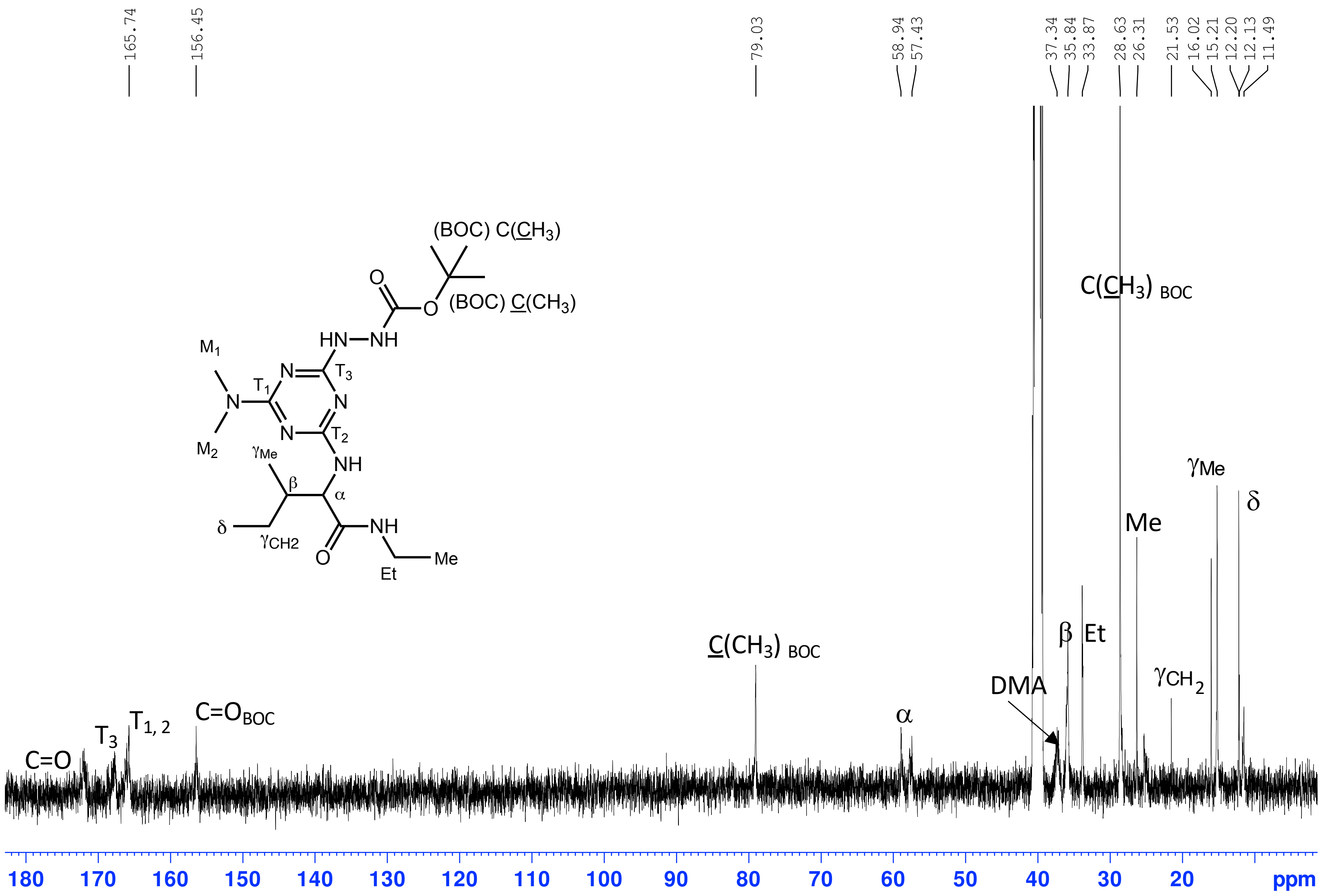

Figure S15. The 400 MHz ^{1}H NMR spectrum of **G^{EA}** in DMSO-d_6. Peaks label (a) originate from excess of **4**.

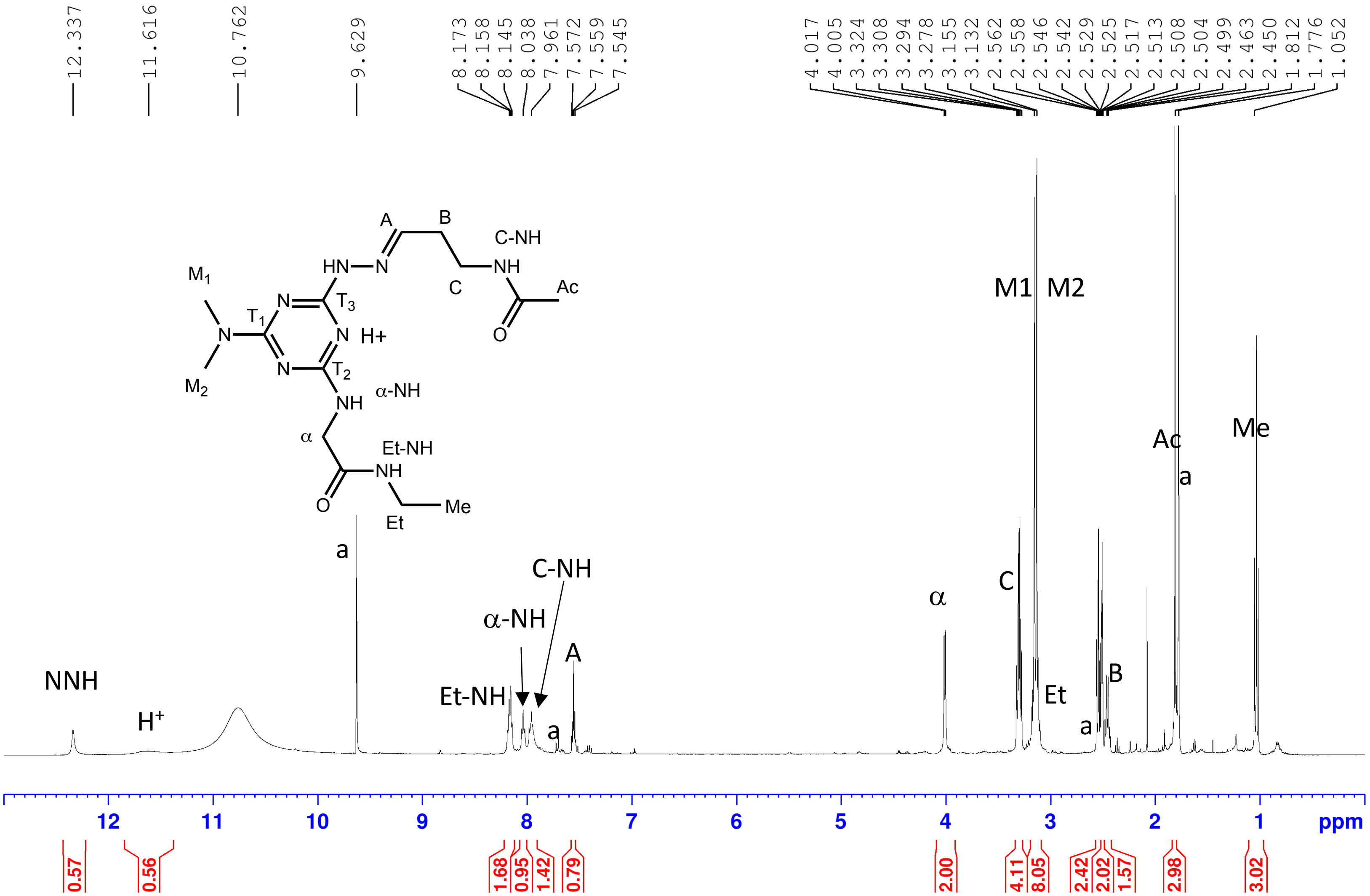

Figure S16. The 100 MHz $^{13}C\{^{1}H\}$ NMR spectrum of $\mathbf{G^{EA}}$ in DMSO-d_6. Peaks label (a) originate from excess of **4**.

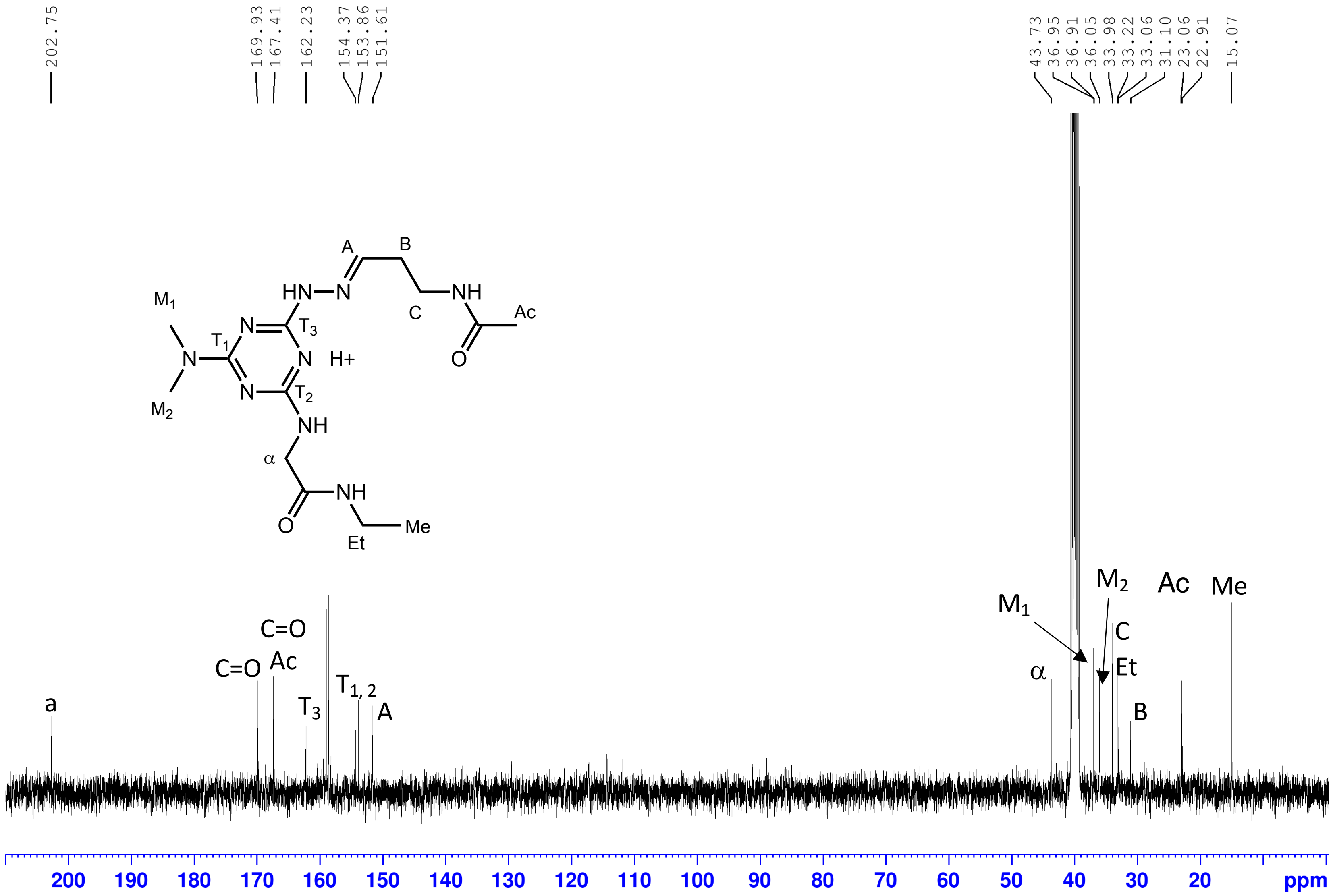

Figure S17. The 400 MHz COSY NMR spectrum of **G^{EA}** in DMSO-d_6.

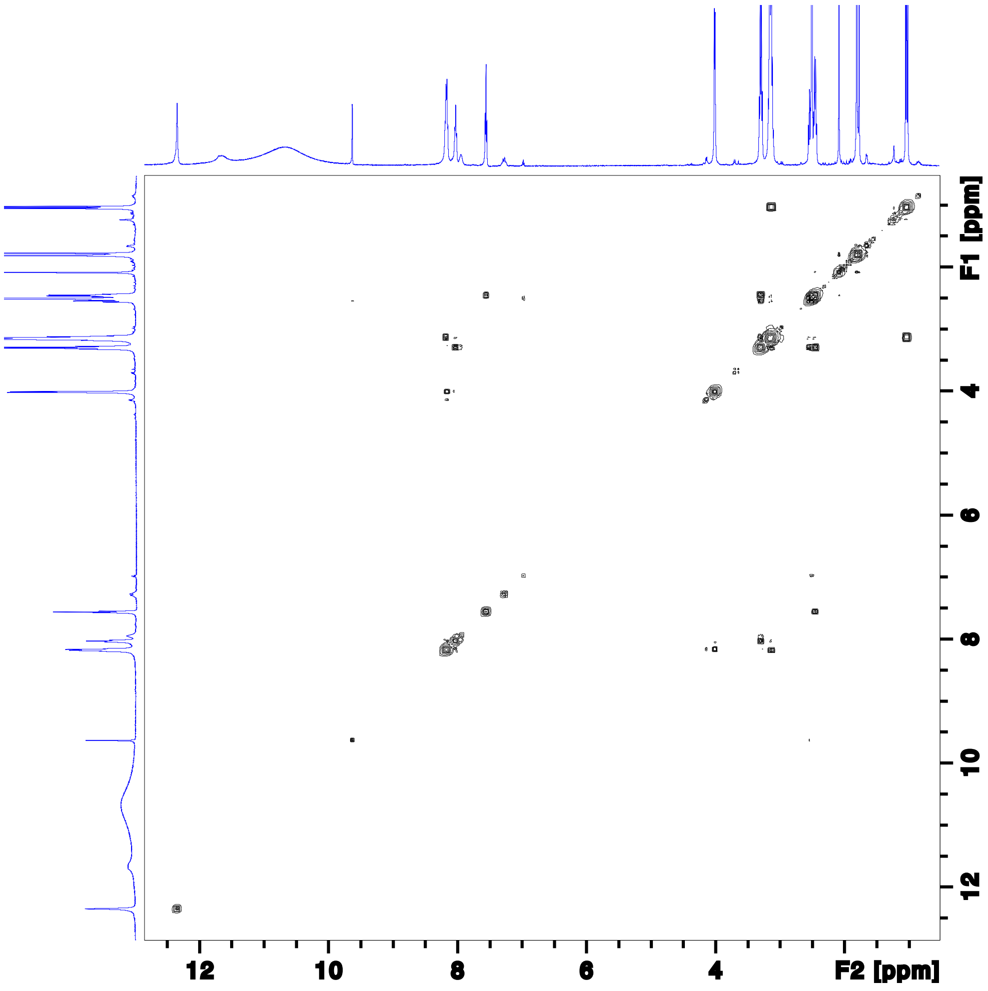

Current Data Parameters
NAME LK-1-47
EXPNO 9
PROCNO 1

F2 - Acquisition Parameters
Date_ 20221122
Time 8.20 h
INSTRUM spect
PROBHD Z108618_0631 (
PULPROG cosygf45
TD 2048
SOLVENT DMSO
NS 8
DS 16
SWH 4934.210 Hz
FIDRES 4.818565 Hz
AQ 0.2075307 sec
RG 203.57
DW 101.333 usec
DE 10.00 usec
TE 298.2 K
D0 0.00000300 sec
D1 2.00000000 sec
IN0 0.00020260 sec
TDav 1
SFO1 400.1326773 MHz
NUC1 1H
P1 10.00 usec
PLW1 23.00000000 W

F1 - Acquisition parameters
TD 128
SFO1 400.1327 MHz
FIDRES 77.122406 Hz
SW 12.335 ppm
FnMODE QF

F2 - Processing parameters
SI 1024
SF 400.1300000 MHz
WDW SINE
SSB 0
LB 0 Hz
GB 0
PC 1.40

F1 - Processing parameters
SI 1024
MC2 QF
SF 400.1300000 MHz
WDW SINE
SSB 0
LB 0 Hz
GB 0

Figure S18. The 400 MHz ^{1}H NMR spectrum of $\mathbf{V^{EA}}$ in DMSO-d_6. Peaks label (a) originate from excess of **4** while (b) originates from diisopropylethyl amine.

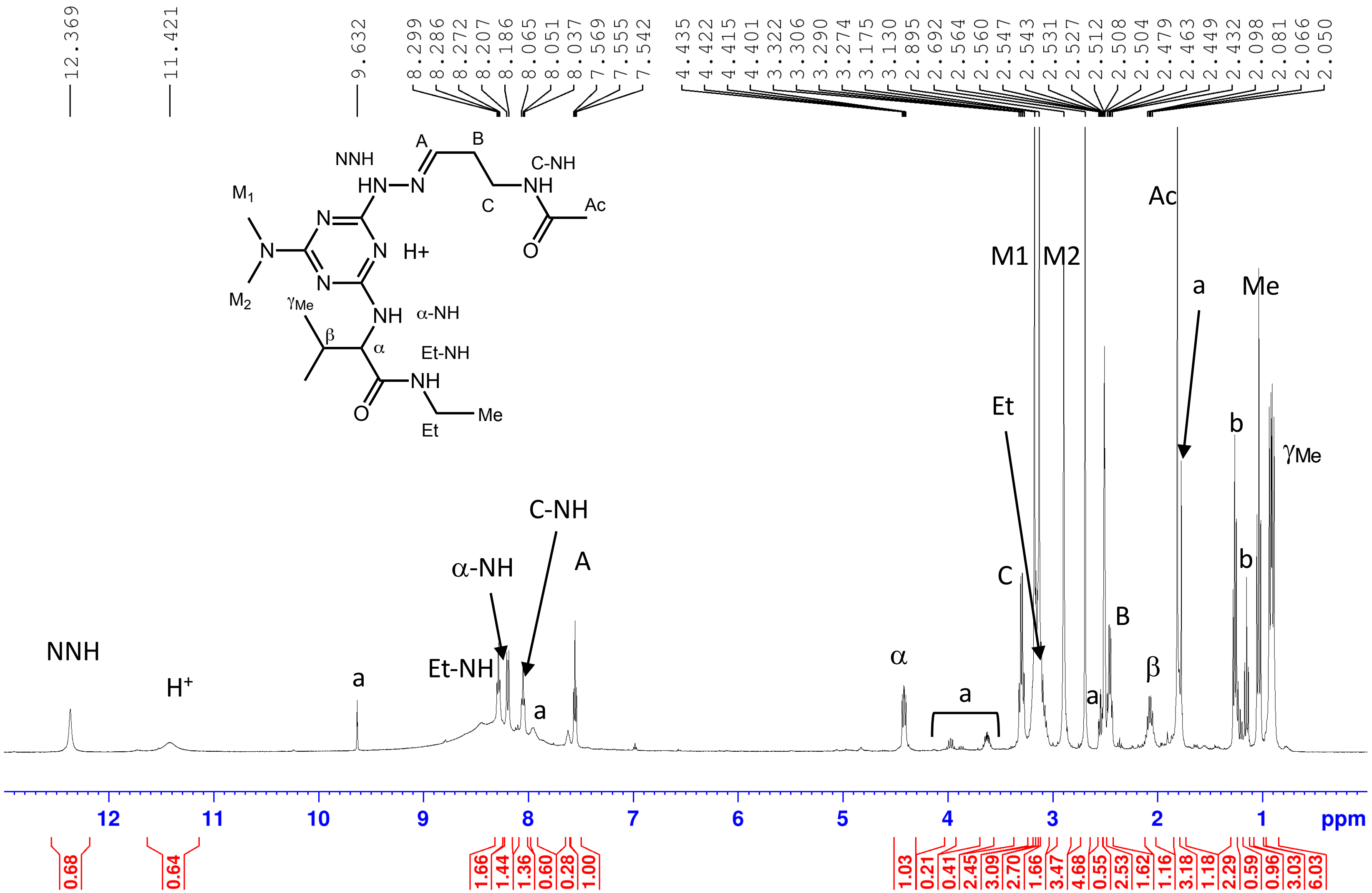

Figure S19. The 100 MHz $^{13}C\{^{1}H\}$ NMR spectrum of $\mathbf{V^{EA}}$ in DMSO-d_6. Peaks labeled (a) originate from excess of **4** while (b) originates from diisopropylethyl amine.

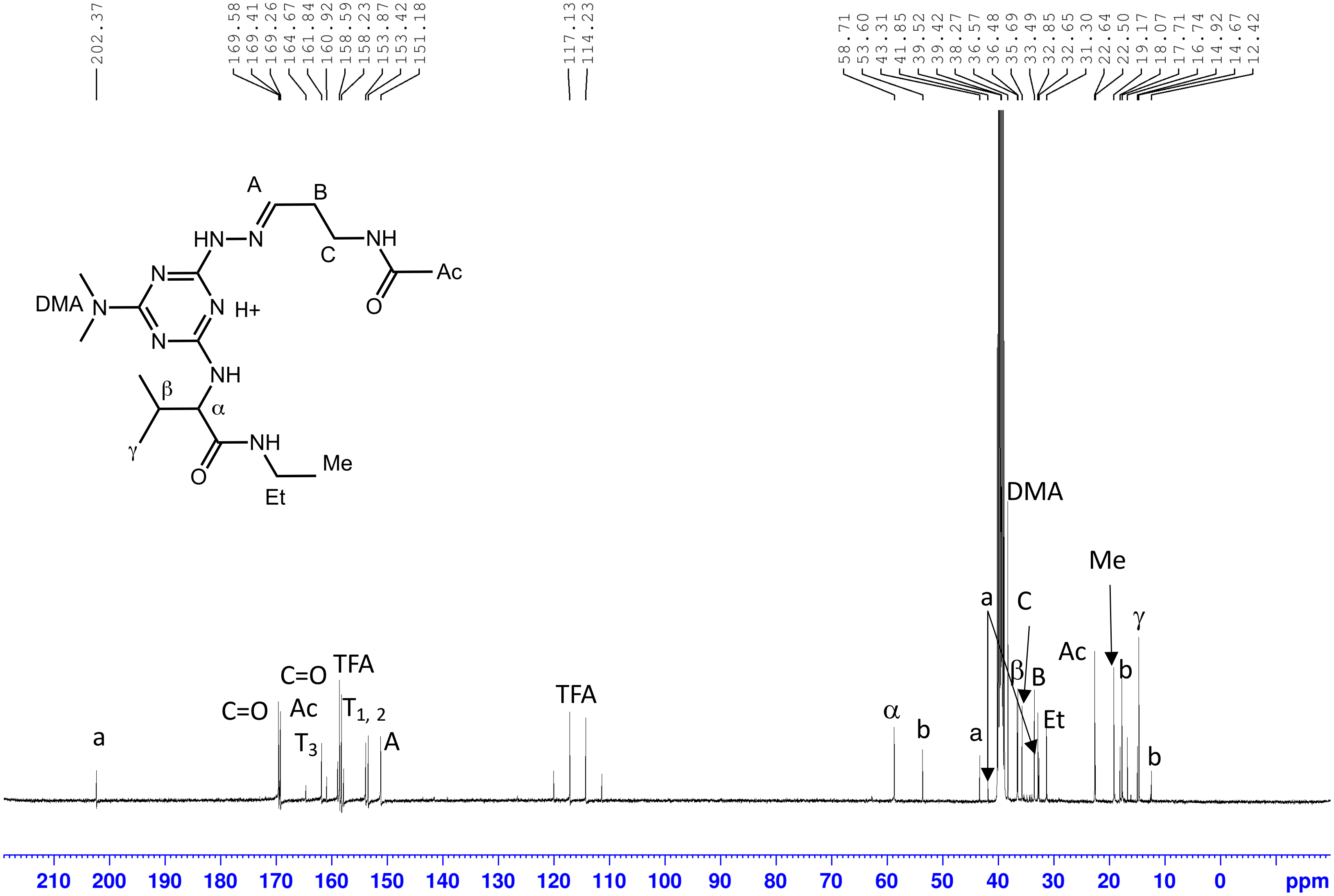

Figure S20. The 400 MHz COSY NMR spectrum of $\mathbf{V^{EA}}$ in DMSO-d_6.

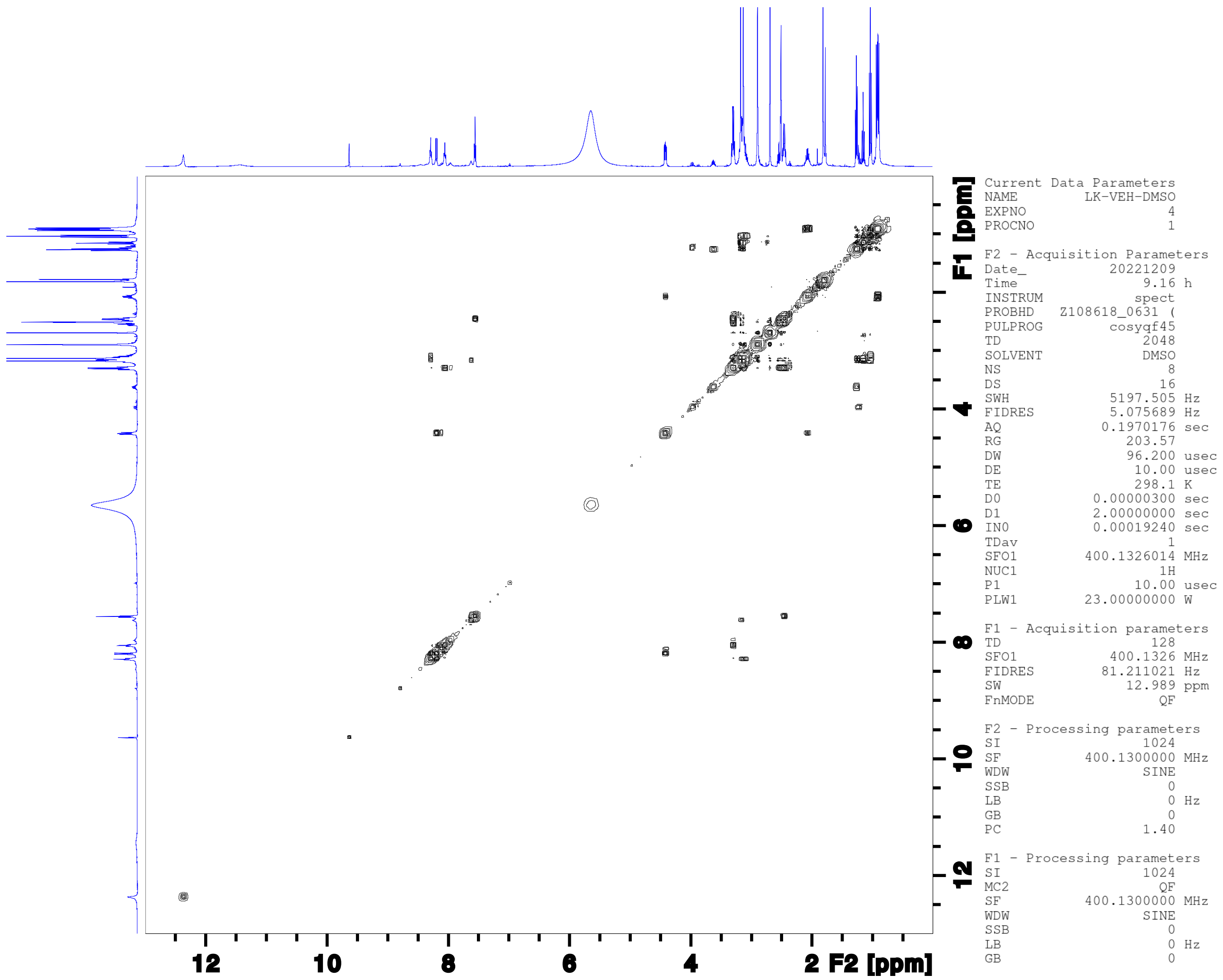

Figure S21. The 400 MHz ^{1}H NMR spectrum of $\mathbf{I^{EA}}$ in DMSO-d_6. Peaks labeled (a) originate from excess of **4** while (b) originates from diisopropylethyl amine.

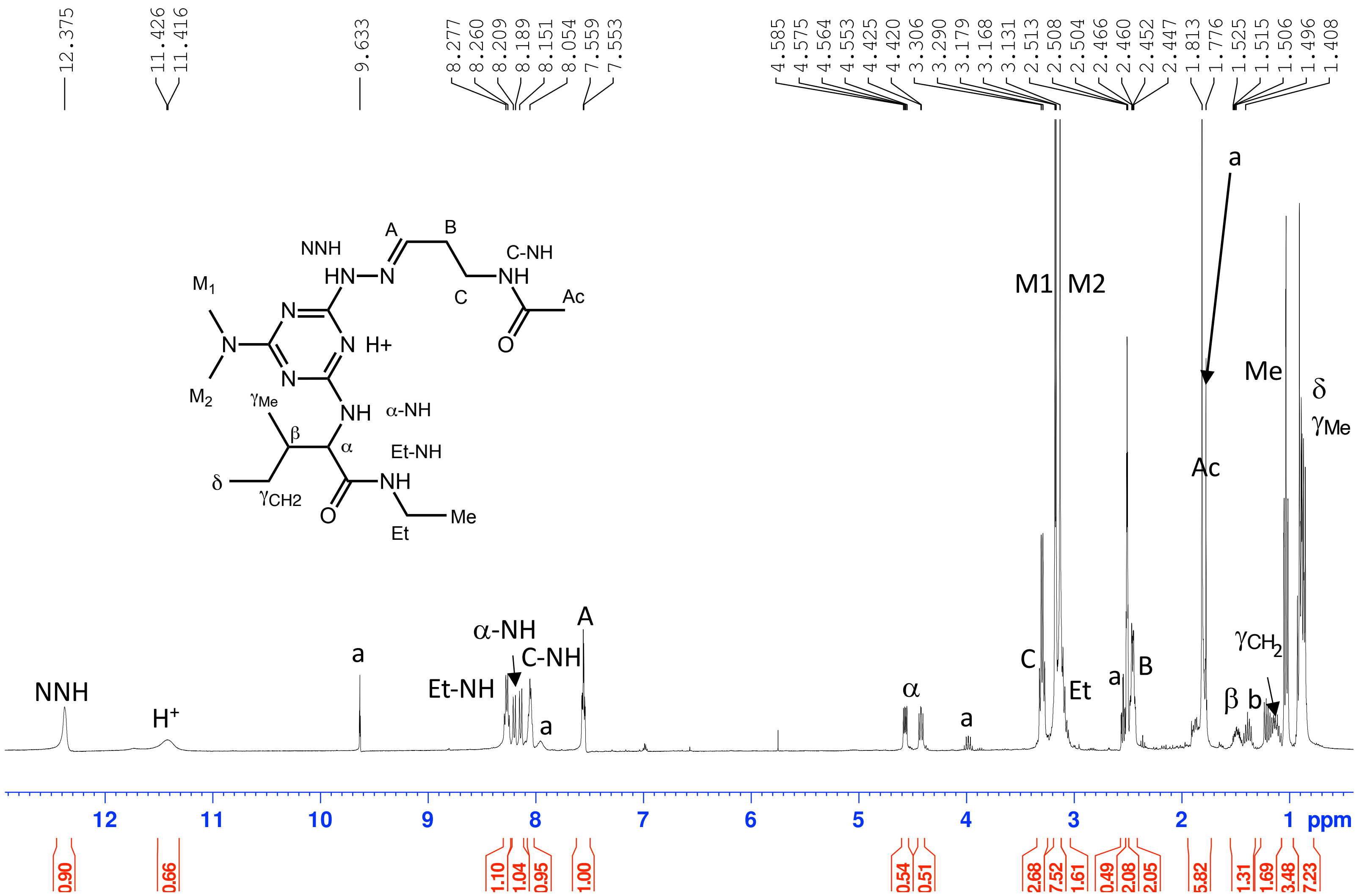

Figure S22. The 100 MHz $^{13}C\{^1H\}$ NMR spectrum of $\mathbf{I^{EA}}$ in DMSO-d_6. Peaks labeled (a) originate from excess of **4**

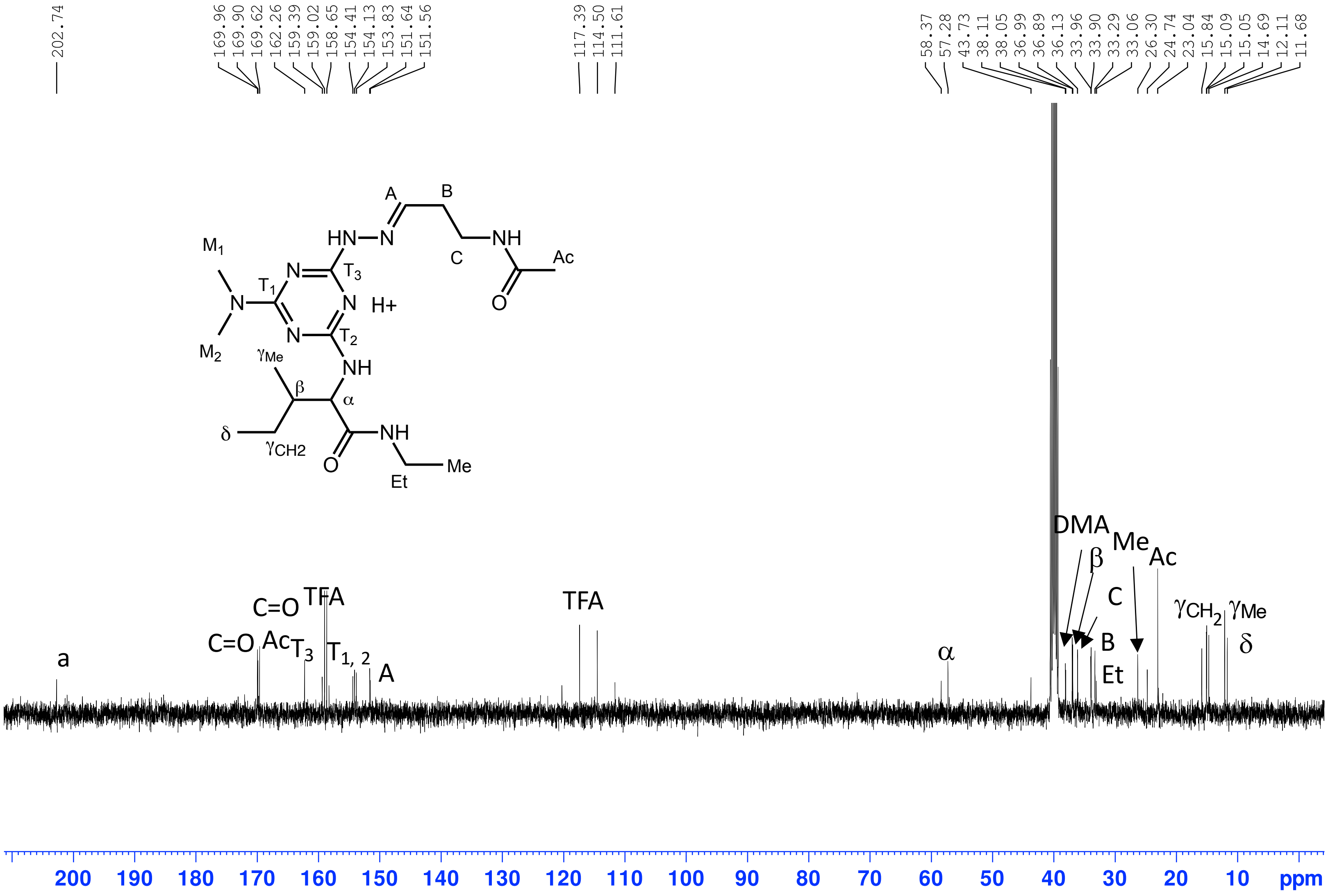

Figure S23. The 400 MHz COSY NMR spectrum of $\mathbf{I}^{\mathbf{EA}}$ in DMSO-d_6.

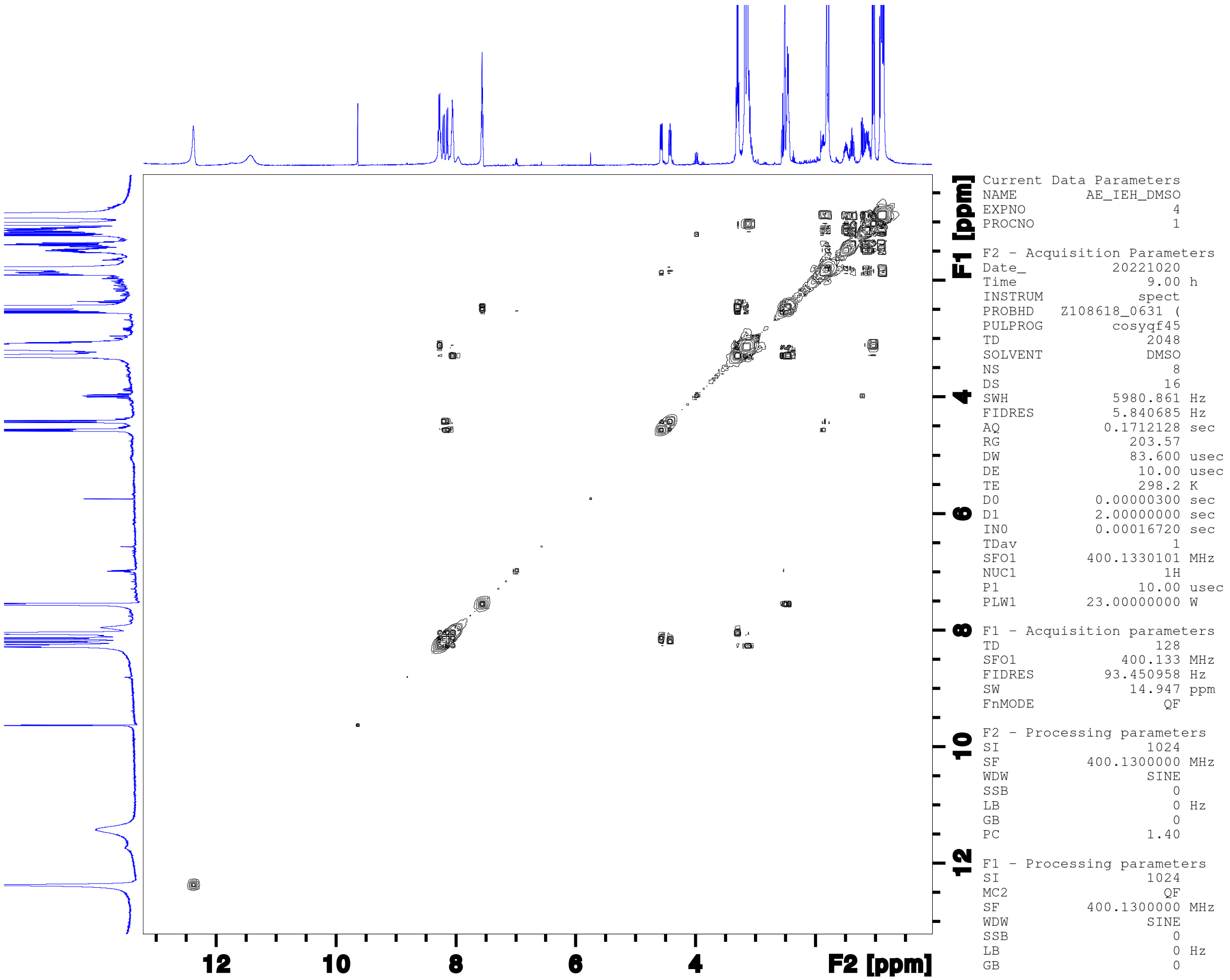

Figure S24. The 400 MHz ^{1}H NMR spectrum of **4** in DMSO-d_6. Note the existence of the deprotected aldehyde.

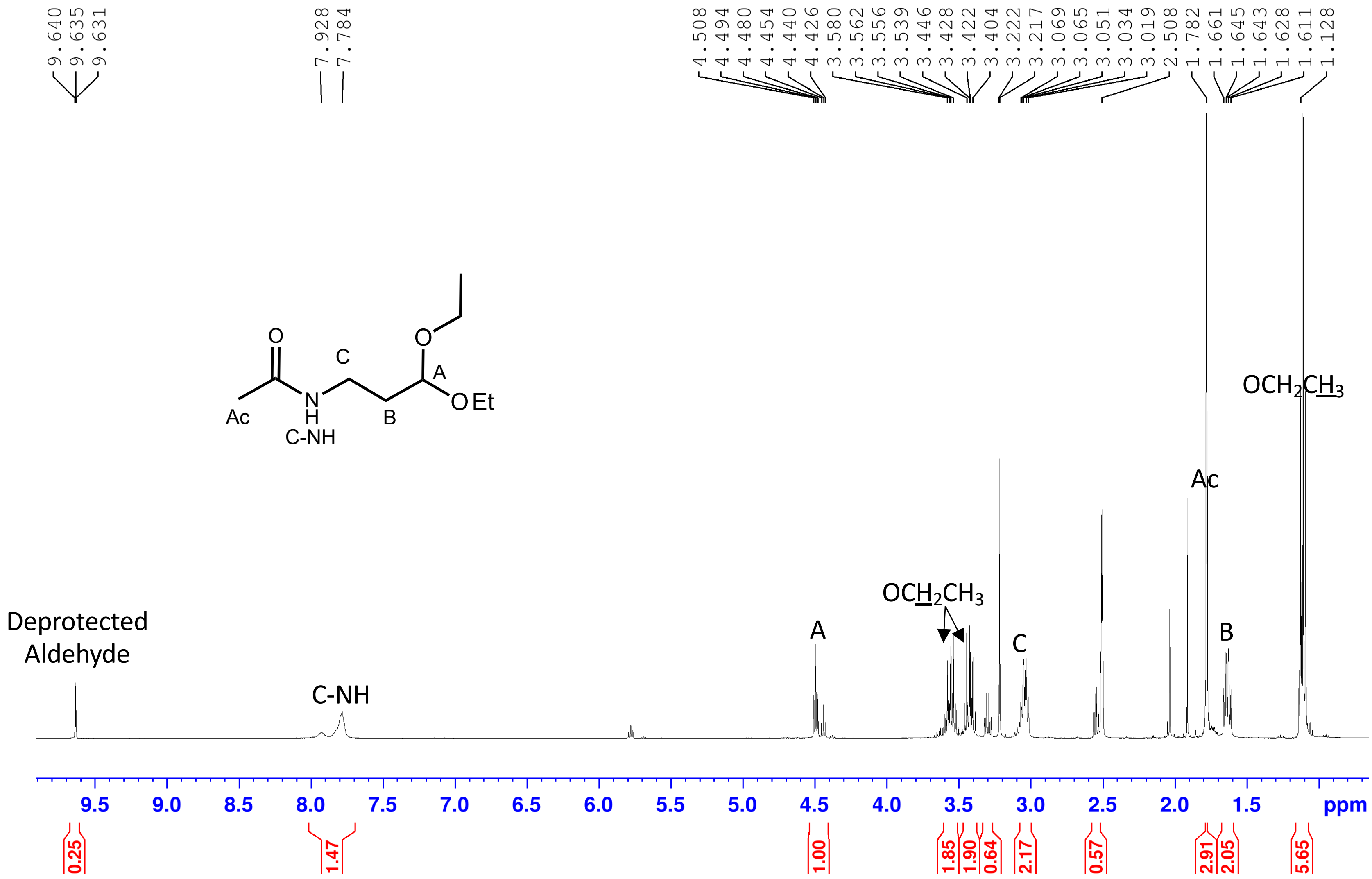

Figure S25. The 100 MHz $^{13}C\{^{1}H\}$ NMR spectrum of **4** in DMSO-d_6. Note the existence of the deprotected aldehyde.

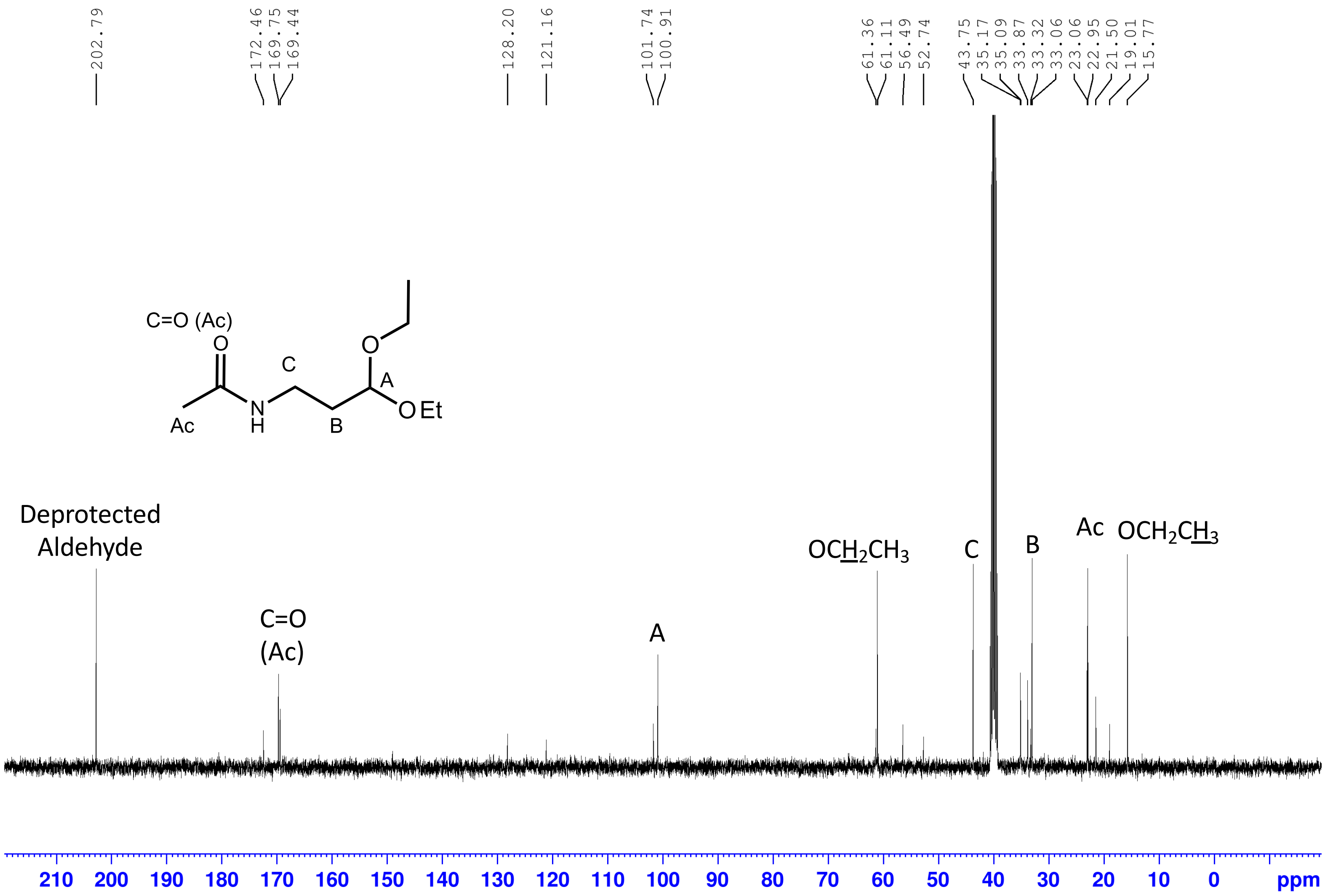

Figure S26. The 400 MHz 1H NMR spectrum of **G-G** in DMSO-d_6.

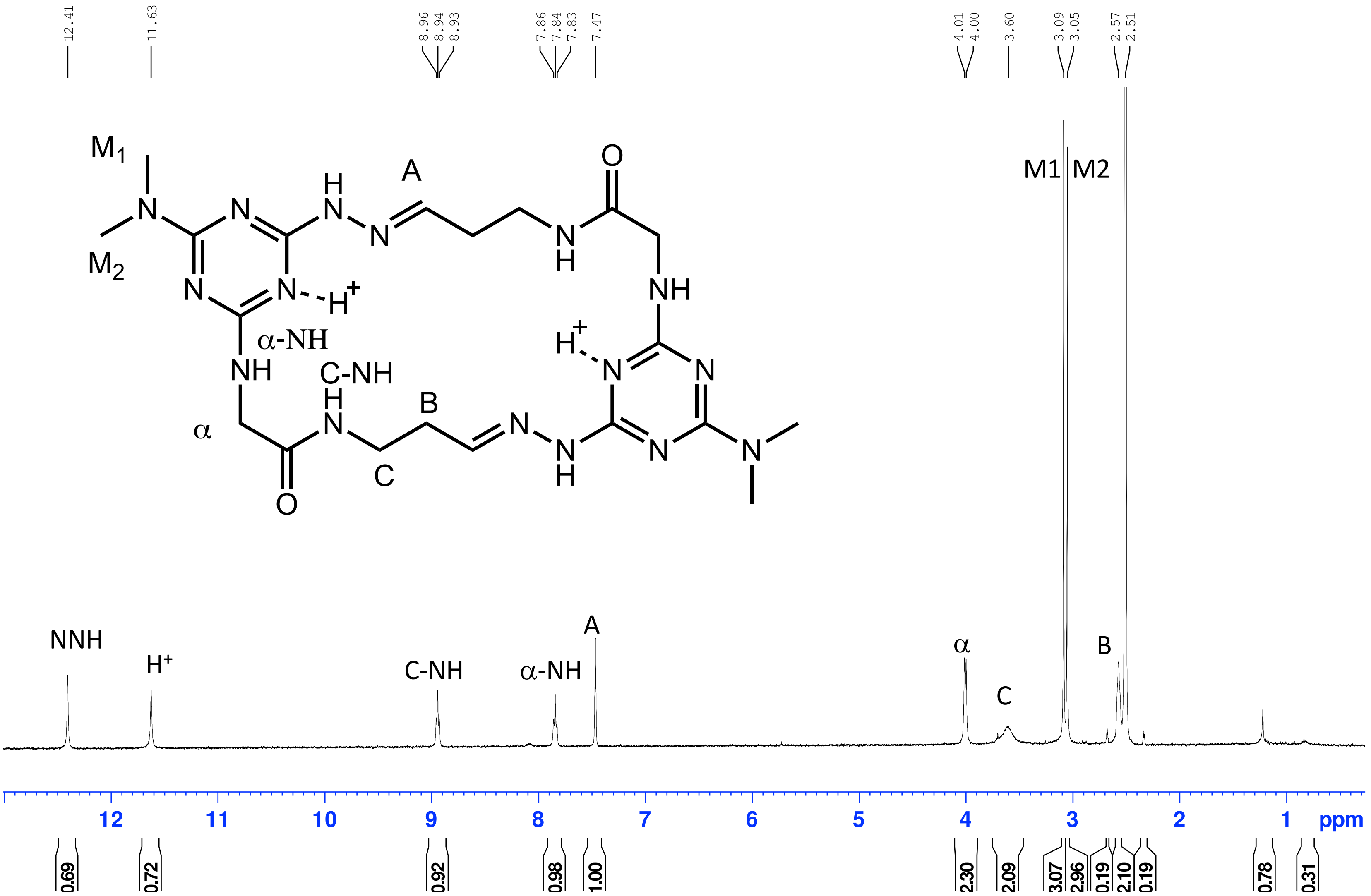

Figure S27. The 400 MHz ^{1}H NMR spectrum of **V-V** in DMSO-d_6.

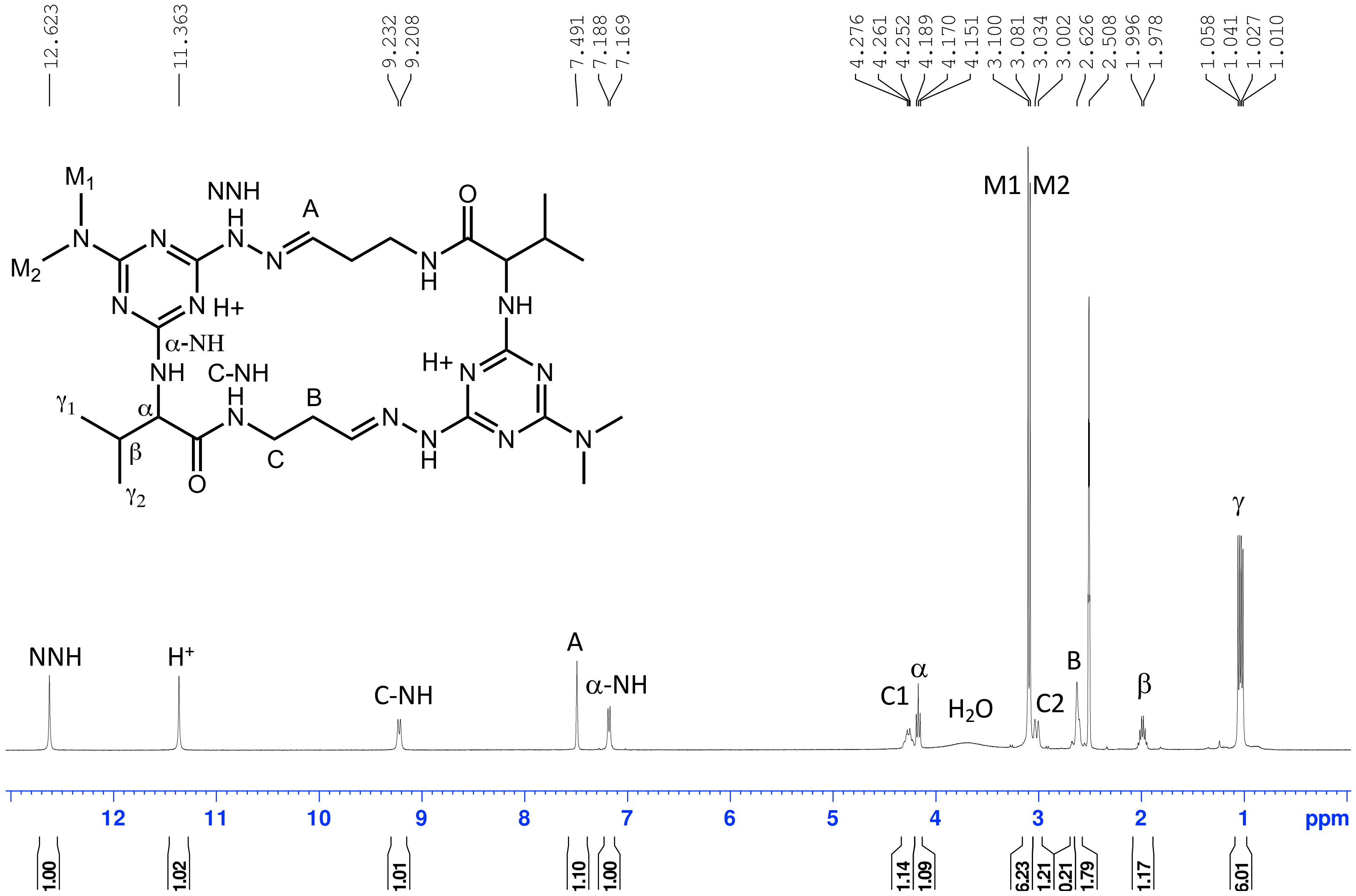

Figure S28. The 400 MHz ^{1}H NMR of **I-I** in DMSO-d_6.

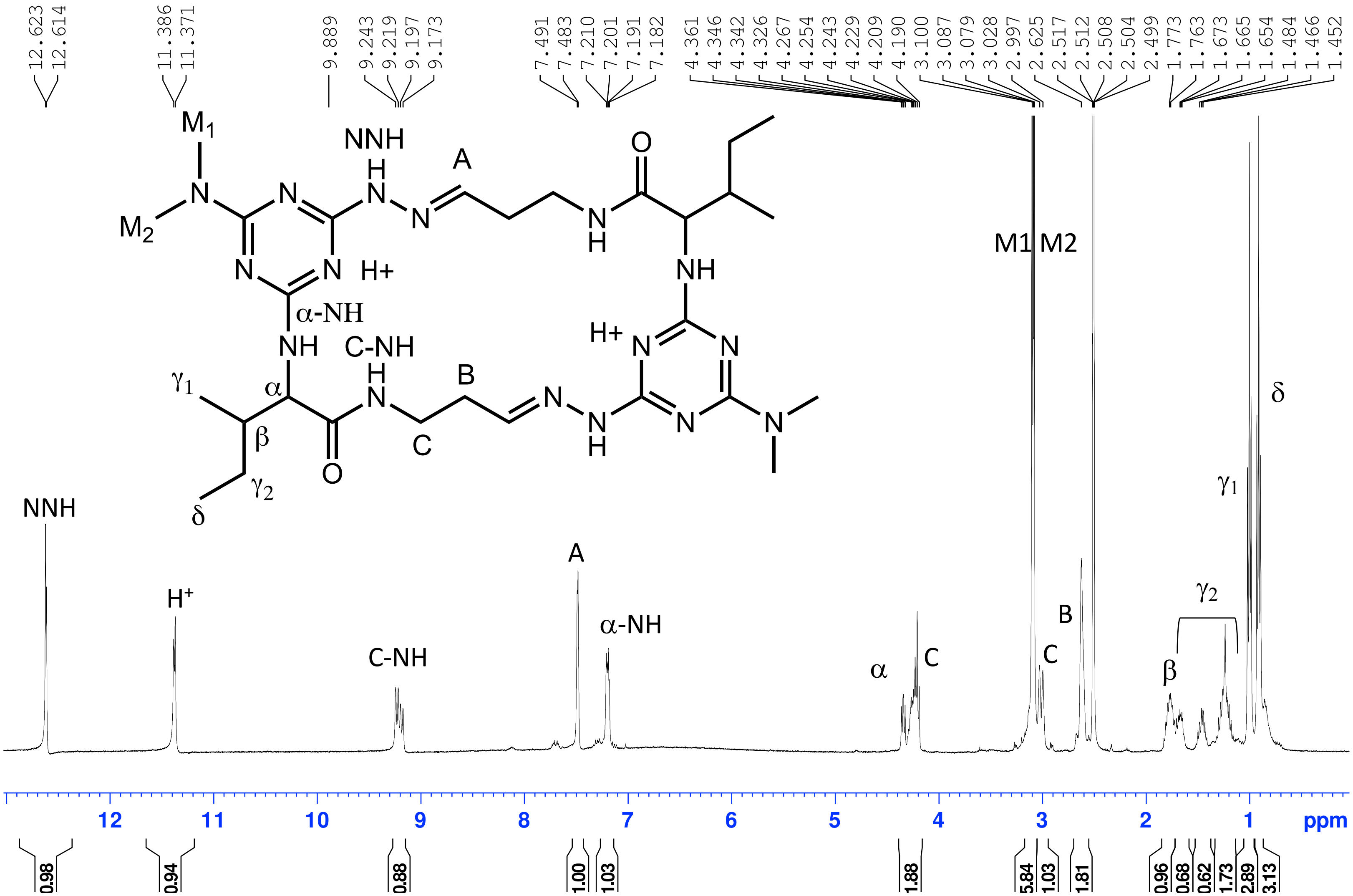

Supplement: Supplementary file 1 [file molecules-28-01144-s001.zip › molecules-2132968-supplementary.pdf]
